# Exploring the Role of Transposable Elements to Sex Gap in Longevity in Three *Drosophila* Species

**DOI:** 10.1111/acel.70788

**Published:** 2026-11-03

**Authors:** Miriam Merenciano, Sonia Janillon, Camille Mermet‐Bouvier, Nelly Burlet, Valentina Rodriguez‐Rada, Victor Ronget, Patricia Gibert, Jean‐Michel Gaillard, Jean‐François Lemaitre, Gabriel A. B. Marais, Matthieu Boulesteix, Marie Fablet, Cristina Vieira

**Affiliations:** ^1^ Laboratoire de Biométrie et Biologie Evolutive, CNRS, UMR5558 Université Claude Bernard Lyon 1 Villeurbanne Rhône‐Alpes France; ^2^ InBIO Laboratório Associado, CIBIO, Centro de Investigação em Biodiversidade e Recursos Genéticos Universidade do Porto Vairão Portugal; ^3^ Departamento de Biologia Faculdade de Ciências, Universidade do Porto Porto Portugal; ^4^ BIOPOLIS Program in Genomics, Biodiversity and Land Planning, CIBIO Vairão Portugal; ^5^ Institut Universitaire de France (IUF) Paris France

**Keywords:** longevity, transcriptome, transposable elements, Y chromosome

## Abstract

In *Drosophila*, like other animal species, females tend to live longer than males, a phenomenon known as sex gap in longevity (SGL). One possible explanation could involve the activity of transposable elements (TE). TEs are normally repressed by epigenetic mechanisms, but this regulation weakens with age. Y chromosomes are enriched in TEs, and age‐related TE activity may therefore be more pronounced in older males, likely affecting longevity (toxic Y hypothesis). Using three *Drosophila* species with different TE content, we showed that SGL varies among wild‐type populations, reflecting both intra‐ and interspecies differences. Transcriptomic data revealed an age‐related increase in TE‐derived reads in *D. melanogaster* and *D. suzukii*, but not in *D. simulans* , with no differences between sexes. However, we observed a higher number of upregulated TE copies in older males compared to older females across the three species tested, which does not necessarily reflect increased transposition activity. Moreover, longevity assays did not reveal an association between increased TE expression and reduced lifespan. Finally, replacing the Y chromosome between strains with different SGL resulted in a progressive and strain‐specific reduction of male lifespan in *D. melanogaster* , despite similar Y chromosome size. This was accompanied by gene expression changes and increased TE expression. In summary, our work calls for a reconsideration of the toxic Y hypothesis, suggesting it may be less relevant in species with low genomic TE content. Notably, Y chromosome effects on lifespan seem to be driven more by Y‐linked heterochromatic polymorphisms and epistatic interactions than by overall TE abundance.

## Graphical abstract

Sex differences in *Drosophila* lifespan are o1en linked to Y‐chromosome TE content. Although TE expression increases with age and a higher number of upregulated TE copies were found in older males, this did not correlate with reduced lifespan. Moreover, Y chromosome replacement affects male longevity and gene and TE expression in *D. melanogaster* , implicating heterochromatic variation and genetic interactions over TE content.

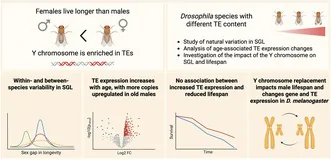

## 1 Introduction

In most animal species, males and females display distinct mortality patterns leading to differences in their longevity (Lemaître, Ronget, and Gaillard 2020; Lemaître, Ronget, Tidière, et al. 2020; Marais et al. 2018). This phenomenon is known as sex gap in longevity (SGL) and several hypotheses have been proposed to explain its evolution across the tree of life (Bronikowski et al. 2022; Brooks and Garratt 2017; Marais et al. 2018). Among these hypotheses, the putative role played by sex‐specific allocation to sexual competition (sexual selection hypothesis) or the asymmetric inheritance of cytoplasmic genomes (mother's curse hypothesis) have been extensively studied (Brooks and Garratt 2017; Camus et al. 2012; Cayuela et al. 2023; Frank and Hurst 1996; Maklakov et al. 2008; Marais et al. 2018; Vinogradov 1998). Sex chromosomes have also been proposed to play an important role in SGL since the heterogametic sex generally has the shortest lifespan (Brown et al. 2020b; Carazo et al. 2016; Marais et al. 2018; Nguyen and Bachtrog 2021; Peona et al. 2021; Pipoly et al. 2015; Xirocostas et al. 2020). On the one hand, the hemizygosity of the X (in XX/XY taxa) or Z (in ZW/ZZ taxa) chromosome suggests that the shortened lifespan of the heterogametic sex might be due to the unconditional expression of recessive deleterious alleles on its single X (or Z) chromosome (unguarded X effect). However, whether this unguarded X effect contributes to sex differences in lifespan is still under debate (Brengdahl et al. 2018; Connallon et al. 2022; Narayan et al. 2022). Alternatively, the toxic Y effect presents another possible hypothesis underlying SGL, postulating that the elevated transposable element (TE) abundance of this chromosome (or W chromosome) compared to other chromosomes may have detrimental consequences for the heterogametic sex with age (Brown et al. 2020b; Nguyen and Bachtrog 2021; Peona et al. 2021; Warmuth et al. 2022). Y (or W) chromosomes often display reduced rates of recombination, resulting in the accumulation of repeated sequences (mainly TEs) on these chromosomes (Erlandsson et al. 2000).

TEs are genomic fragments with the ability to move along the genome, representing a major component of eukaryotic genomes (Bourque et al. 2018). Due to their mobile and repetitive nature, they are considered a source of mutations and genetic polymorphisms (Bourque et al. 2018). As such, TE activity (transcription and transposition) is normally repressed by epigenetic regulation that involves DNA methylation, histone modifications and/or small RNAs (Merenciano et al. 2024; Slotkin and Martienssen 2007). With age, these silencing mechanisms are disrupted causing a general increase of TE activity (Copley and Shorter 2023; Merenciano et al. 2025; Wood and Helfand 2013; Yushkova and Moskalev 2023). The toxic Y effect hypothesis thus proposes that because of the high abundance of TEs on the Y chromosome, more TEs may become active in older males than in older females, which might contribute to sex differences in aging and in the reduced male lifespan (Marais et al. 2018).

Age‐dependent activation of TEs has been suggested to negatively impact lifespan in *Drosophila* (Brown et al. 2020b; Chen et al. 2016; Driver and McKechnie 1992; Fabian et al. 2021; Li et al. 2013; Nguyen and Bachtrog 2021; Nikitin and Woodruff 1995; Rigal et al. 2022; Wood et al. 2016; Wood and Helfand 2013; Woodruff and Nikitin 1995; Yang et al. 2022), termites (Elsner et al. 2018; Post et al. 2023), nematodes (Sturm et al. 2023), mice (De Cecco et al. 2019; Ricci et al. 2023), and humans (Bogu et al. 2019; Teoli et al. 2025; Tsai et al. 2024; Van Meter et al. 2014). More specifically, in *Drosophila melanogaster* , it has been suggested that aged males undergo a general loss of H3K9me2 histone modification more rapidly than females, resulting in a more pronounced TE expression during aging in males (Brown et al. 2020a). Indeed, in this species, it was described that the Y chromosome, and TEs in general, affect chromatin formation genome‐wide, in agreement with the heterochromatin sink model (Brown et al. 2020a; Gatti and Pimpinelli 1992). In addition, longevity assays performed in flies with aberrant sex chromosome configurations (XO, XXY, and XYY) showed that the number of Y chromosomes could influence organismal survival (Brown et al. 2020b).

In other studies, the above conclusions have been challenged. For instance, the generation of *D. melanogaster* Y chromosomes with different sizes using CRISPR/Cas9 suggested that the size of this chromosome, and thus, the amount of heterochromatin (and TEs) did not contribute to sex‐specific differences in longevity (Delanoue et al. 2023). In *D. pseudoobscura* , variations in Y chromosome size and Y‐linked TEs showed that larger Y chromosomes lead to slightly increased TE expression, especially in old males, but these differences do not result in faster aging (Nguyen et al. 2022). Therefore, the impact of the Y chromosome on male longevity through TE abundance and increased TE activity is still under debate.

In this work, we studied natural variation in SGL across three *Drosophila* species with varying TE content: *D. melanogaster* , *D. simulans* , and *D. suzukii*. Additionally, we analyzed age‐associated TE expression changes using RNA‐seq, with particular emphasis on potential sex‐specific differences. This approach measures TE transcriptional activity, but does not allow direct assessment of TE mobilization. Finally, we performed Y chromosome replacement experiments to investigate the impact of the Y chromosome on SGL and lifespan.

## 2 Results

### 2.1 SGL Varies Between Populations and Species

To study the natural variation in SGL, we performed longevity assays in 277 strains from three *Drosophila* species: *D. melanogaster* , *D. simulans* , and *D. suzukii*. For each species, we analyzed between two and six wild‐type populations, totaling 28–132 strains, with 100 flies per strain (Figure 1 and Table S1). SGL was measured as the natural logarithm of the ratio between male and female median lifespan for each strain, a metric previously used in mammals (see Section 4) (Table S2) (Lemaître, Ronget, and Gaillard 2020; Lemaître, Ronget, Tidière, et al. 2020).

**FIGURE 1:**
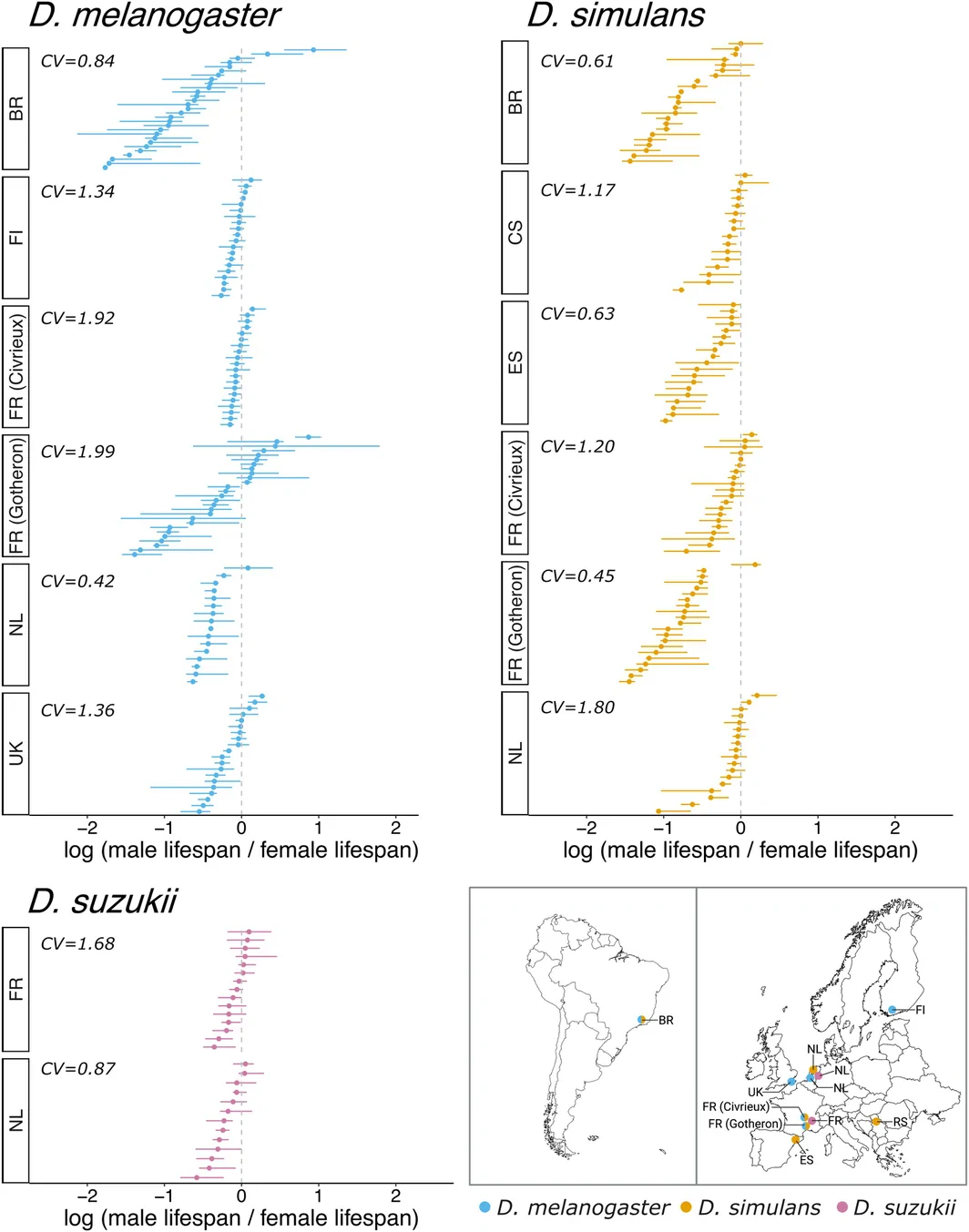
SGL values per strain and geographical distribution of all the populations tested from *D. melanogaster* , *D. simulans* , and *D. suzukii*. Dots show mean SGL values calculated as the natural logarithm of the ratio between male and female median lifespan, and lower and upper whiskers indicate the upper and lower 95% confidence intervals. Coefficients of variation (CV) were calculated for every population. UK: United Kingdom, FR: France, FI: Finland, NL: The Netherlands, BR: Brazil, ES: Spain, RS: Serbia.

59% of strains across all species exhibited significantly negative SGL values, indicating reduced male longevity, while only 5% showed significantly positive values, indicative of reduced female longevity (Figure 1 and Table S2). Specifically, statistically significant negative SGL values were observed in 58% of *D. melanogaster* strains, 65% of *D. simulans* strains, and 35% of *D. suzukii* strains (Figure 1 and Table S2). On average, male lifespan was shorter by 10.5 days in *D. melanogaster* , 12.5 days in *D. simulans* , and 9 days in *D. suzukii*.

We then analyzed the variance in the SGL variable across different hierarchical levels (population and species) using a Bayesian approach (see Section 4). The analysis revealed that the population of origin contributed significantly to the observed differences in SGL (Figure 1 and Figure S1A). Notably, it explained 38.2% (95% CI [16.2, 57.5]) of the total variance, suggesting substantial within‐species variability attributable to population‐level factors. In *D. melanogaster* , the coefficient of variation in SGL values ranged from 0.42 to 1.99, while in *D. simulans* , it ranged from 0.45 to 1.80 (Figure 1). For *D. suzukii*, the coefficients ranged from 0.87 to 1.68 (Figure 1). The species effect explained 8.6% (95% CI [0.1, 20.8]) of the total variance, also suggesting differences in SGL across species (Figure 1 and Figure S1B).

In summary, our extensive analysis of SGL showed that males have an overall reduced lifespan compared to females in all three *Drosophila* species tested. Moreover, we found significant within‐ and between‐species variability of SGL values.

### 2.2 *D. melanogaster* and *D. suzukii* Flies Show Higher Abundance of Expressed TE Families With Age

To study TE expression changes associated with age, we used the same three related *Drosophila* species, selected because of their varying TE content: *D. melanogaster* (20%), *D. simulans* (12%), and *D. suzukii* (47%) (Mérel et al. 2020, 2021). Comparing species with such divergent TE content enabled us to test whether age‐related TE expression changes are influenced by overall genomic TE content. RNA‐seq data were obtained from young and old individuals of two inbred strains per species, whose genome was previously sequenced using long‐read techniques: dmgoth101 and dmgoth63 for *D. melanogaster* , dsgoth31 and dsgoth613 for *D. simulans* , and MT47 and S29 for *D. suzukii* (see Section 4) (Table S1 and Figure S2) (Mohamed et al. 2020).

A substantial proportion of genes previously associated with aging were differentially expressed in older flies: 14.1%–24.2% in *D. melanogaster* males, 44.3%–45.0% in females; 27.0%–29.6% in *D. simulans* males, 49.3%–53.9% in females; and 25.8%–40.8% in *D. suzukii* males, 25.8%–30.0% in females (Figures S3 and S4). This indicates that sampling age captured a biologically meaningful aging stage, despite variations in chronological age across strains and species (see Section 4).

We then used *TEtools* to assess whether TE expression varies with age, irrespective of sex (see Section 4) (Tables S3 and S4) (Lerat et al. 2017). To do that, we first looked at the percentage of reads mapping to TE sequences in our RNA‐seq data. Using a generalized linear model (GLM) analysis (see Section 4), we found that the percentage of TE‐derived reads increased in older flies in all the species tested (estimate 0.314, *p* < 0.001) (Figure 2A). In particular, *D. melanogaster* showed a greater difference between young and old flies compared to the other species (Figure 2A). Furthermore, compared to *D. melanogaster* , *D. simulans* exhibited fewer TE‐derived reads (estimate −0.652, *p* < 0.001), whereas *D. suzukii* showed more (estimate 0.725, *p* < 0.001), consistent with their respective TE contents (Figure 2A).

**FIGURE 2:**
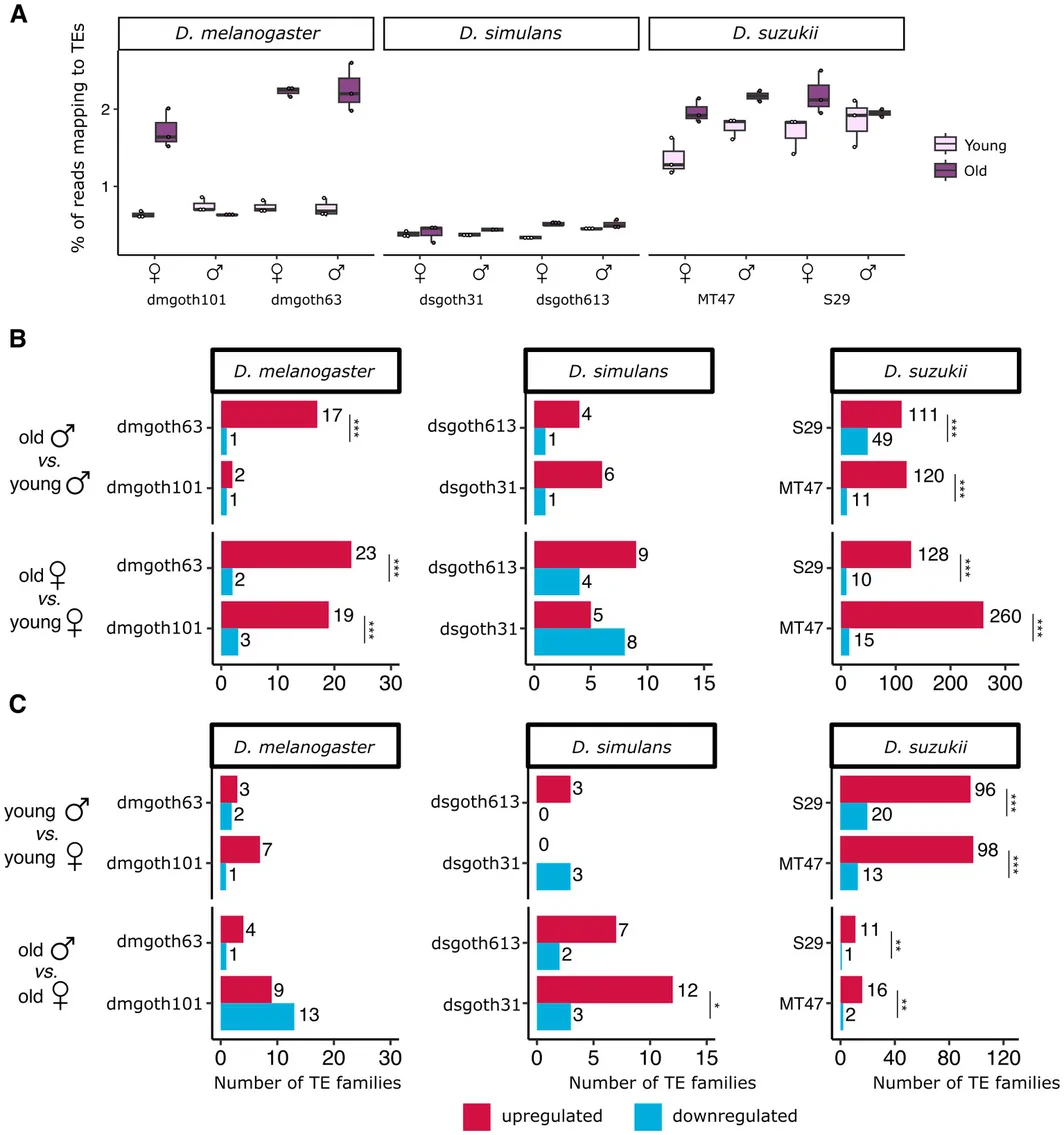
Aging increases the number of TE transcripts and upregulated TE families in different *Drosophila* species. (A) Boxplots showing the percentage of reads mapping to TE sequences in the RNA‐seq data in young and old male and female flies of each strain. Boxplots show the median (horizontal line), first and third quartiles (lower and upper bounds, respectively), and minimum and maximum values (lower and upper whiskers, respectively). Each dot represents an independent biological replicate. (B) Barplots showing the number of upregulated (in red) and downregulated (in blue) TE families with aging for both males (upper panel) and females (bottom panel) in each strain. (C) Barplots showing the number of upregulated (in red) and downregulated (in blue) TE families in young males compared to young females (upper panel) and in old males compared to old females (bottom panel) in each strain. TE families were considered as significantly differentially expressed when the adjusted *p*‐value was < 0.05 and the absolute log2 fold‐change > 1. TE families with a log2 fold change > 1 were classified as upregulated, whereas those with a log2 fold change < −1 were classified as downregulated. Asterisks indicate level of statistical significance of one‐sided binomial tests (*p ≤ 0.05, **p ≤ 0.01, ***p ≤ 0.001).

Since certain TE families might contribute more to the age‐related increase in expression, we also analyzed TE expression at the family level (Figure S5 and Tables S3–S7). In both *D. melanogaster* and *D. suzukii*, we observed a greater number of upregulated TE families compared to downregulated ones in older flies across all strains (one‐sided binomial test, all *p* < 0.001), except for males from the *D. melanogaster* dmgoth101 strain (one‐sided binomial test, *p* = 0.545) (Figure 2B). Similar results were found for both males and females, suggesting that TE transcription increased with aging as has been observed in other studies from *D. melanogaster* and other species, such as humans (Figure 2B) (Bogu et al. 2019; Brown et al. 2020b; Chen et al. 2016; Driver and McKechnie 1992; Fabian et al. 2021; Li et al. 2013; Nguyen and Bachtrog 2021; Nikitin and Woodruff 1995; Rigal et al. 2022; Teoli et al. 2025; Tsai et al. 2024; Van Meter et al. 2014; Wood et al. 2016; Woodruff and Nikitin 1995; Yang et al. 2022). However, in *D. simulans* , the number of upregulated TE families did not statistically differ from the number of downregulated ones in older flies (one‐sided binomial test, all *p* > 0.05), probably due to its reduced TE content. Nonetheless, there was a tendency towards a higher number of upregulated TE families (Figure 2B). Although the number of upregulated TE families generally increased with age, these TE families showed little to no overlap between sexes or between strains of the same species (Figure S6). This result suggests that TE expression during aging is not family specific.

Overall, we observed an increased number of upregulated TE families in older flies of *D. melanogaster* and *D. suzukii*. In *D. simulans* , there was a similar tendency, although it was not statistically significant, likely attributed to its reduced TE content. These findings are in line with the hypothesis that aging is associated with the disruption of the epigenetic mechanisms that silence TEs, leading to their activation. As we assessed TE activation using RNA‐seq, we quantified transcriptional expression rather than new insertion events. Although TE expression can be a prerequisite for mobilization, increased expression does not necessarily imply that mobilization occurred.

### 2.3 The Age‐Related Increase in TE‐Derived Reads and Expressed TE Families Is Not Influenced by Sex

One expectation of the toxic Y chromosome hypothesis is that older males, which harbor TE‐enriched Y chromosomes, should have increased TE expression levels. We investigated this by comparing the percentage of TE‐derived reads between older males and older females.

Performing a GLM analysis, we found no statistically significant effects of sex (estimate 0.056, *p* = 0.398) or the interaction between sex and age (estimate −0.140, *p* = 0.271) on the percentage of TE‐derived reads (Figure 2A). Similarly, at the TE family level, we found no statistically significant differences between the number of upregulated and downregulated TE families when comparing older males with older females, except in the *D. simulans* dsgoth31 strain and the *D. suzukii* S29 and MT47 strains (one‐sided binomial test, *p* = 0.035, *p* = 0.009, and *p* = 0.004, respectively) (Figure 2C and Figure S7). Again, little overlap between differentially expressed TE families was found between strains from the same species, suggesting that there are no particular TE families leading to the sex differences in TE expression of older flies (Figure S6). When comparing the expression of TE families between young male and young female flies, we observed no statistically significant differences between the number of upregulated and downregulated TE families in *D. melanogaster* and *D. simulans* (one‐sided binomial test, all *p* > 0.05) (Figure 2C). Conversely, in *D. suzukii*, we did observe more upregulated TE families than downregulated ones when comparing young males and young female flies (one‐sided binomial test, *p* < 0.001) (Figure 2C).

Altogether, we found no significant sex differences in the overall age‐related increase of TE‐derived reads. Comparisons between older males and older females revealed more upregulated TE families than downregulated ones only in one *D. simulans* strain and in both *D. suzukii* strains.

### 2.4 *D. melanogaster* and *D. suzukii* Flies Show Higher Abundance of Expressed TE Copies With Age

We previously observed that TE‐derived reads, as well as some TE families, increased their expression with age, without major sex‐specific effects. However, our family‐level approach might mask differences in the expression of individual TE copies, which could be selectively expressed or released due to locus‐specific chromatin relaxation. Throughout the text, the term “TE copies” is then used to refer to individual genomic TE loci. To address this, we performed a copy‐specific TE expression approach by using *Telescope* software, which measures TE expression at specific genomic locations, allowing us to assess the impact of age and sex on individual TE copies (see Section 4) (Tables S3, S4, and S8–S13) (Bendall et al. 2019). *Telescope* is a computational tool that resolves ambiguous mapped reads by probabilistically assigning them to their most likely source transcript using a Bayesian model (Bendall et al. 2019).

On the one hand, we found a general trend towards more upregulated than downregulated TE copies with age in both males and females of *D. melanogaster* and *D. suzukii*, confirming our previous analysis showing that the percentage of TE‐derived reads increased in older flies (Figure 3A and Figure S8). These results were statistically significant (one‐sided binomial test, all *p* < 0.05) (Figure 3A). On the other hand, in *D. simulans* , the number of upregulated TE copies in older flies did not differ significantly from the number of downregulated TE copies when compared with young flies (one‐sided binomial test, all *p* > 0.05) (Figure 3A). These findings are in line with our previous analysis on TE‐derived reads analyzed using *TEtools* software (Figure 2A). Moreover, as expected based on the TE content, *D. suzukii* showed a higher number of differentially expressed TE copies (DETEs) in both males and females compared to the other species (Figure 3A).

**FIGURE 3:**
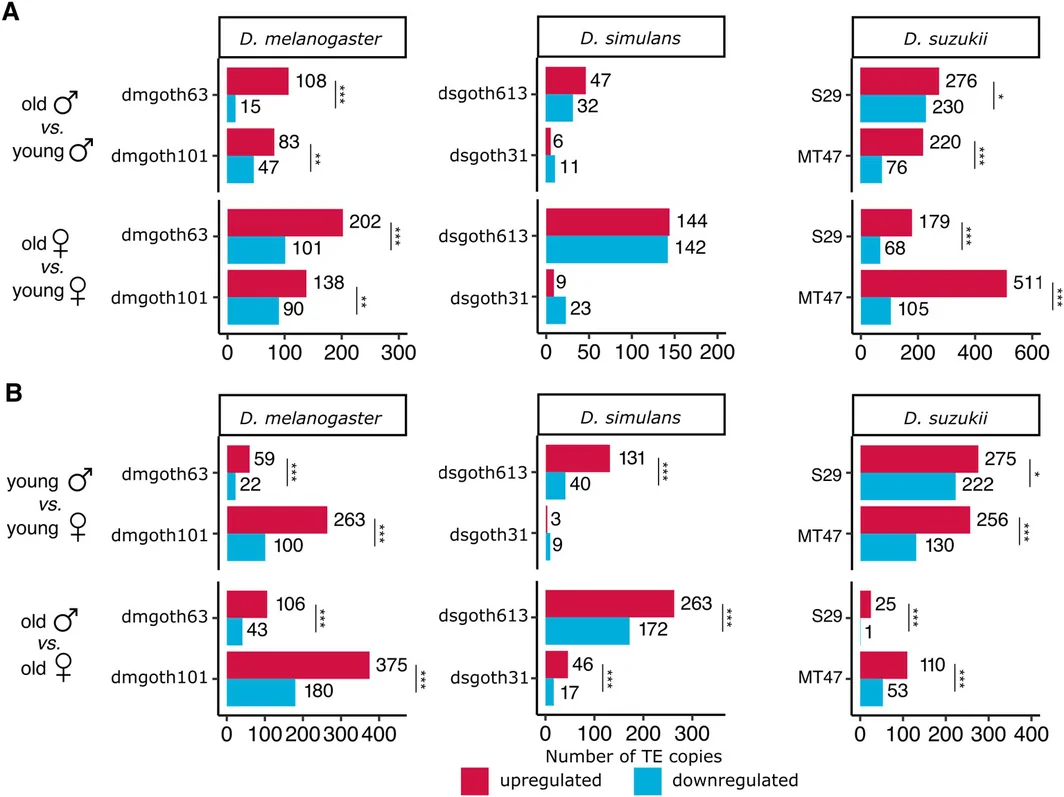
Aging increases the number of activated TE copies in different *Drosophila* species. (A) Barplots showing the number of upregulated (in red) and downregulated (in blue) TE copies with aging for both males (upper panel) and females (bottom panel) in each strain. (B) Barplots showing the number of upregulated (in red) and downregulated (in blue) TE copies in young males compared to young females (upper panel) and in old males compared to old females (bottom panel) in each strain. TE copies were considered as significantly differentially expressed when the adjusted *p*‐value was < 0.05 and the absolute log2 fold‐change > 1. TE copies with a log2 fold change > 1 were classified as upregulated, whereas those with a log2 fold change < −1 were classified as downregulated. Asterisks indicate level of statistical significance of one‐sided binomial tests (*p ≤ 0.05, **p ≤ 0.01, ***p ≤ 0.001).

We also tested whether male and female flies from the same strain shared some of the DETEs. Indeed, we found that some of the upregulated DETEs identified in males were also identified in females (Figure S9). These shared DETEs are therefore absent from the Y chromosome, indicating that a subset of activated TEs might not be present in the Y chromosome, at least in some strains, suggesting a broader pattern of TE expression.

In summary, we observed an increased number of upregulated TE copies compared to downregulated ones in older flies of *D. melanogaster* and *D. suzukii*, but not in *D. simulans* .

### 2.5 More TE Copies Are Expressed in Older Males Compared to Older Females

Using the same copy‐specific TE expression approach, we tested whether there were sex differences in the expression of TE copies with age.

We detected more TE copies with increased than decreased expression in older males relative to older females in all the strains of the three species tested (one‐sided binomial test, all *p* < 0.05) (Figure 3B and Figure S10). These differences were already observed at young ages (one‐sided binomial test, all *p* < 0.05), with the exception of the *D. simulans* dsgoth31 strain (one‐sided binomial test, *p* = 0.981) (Figure 3B). In contrast to the other two species, *D. suzukii* showed a greater number of DETEs between sexes in young flies than in older flies (Figure 3B).

We also found that the upregulated TE copies observed between older males and older females were present in all chromosome arms, with an expected concentration in chromosome centromeres and in the dot chromosome (Figure S11). In all strains, full‐length TE copies, which have greater potential to transpose, were significantly overrepresented among upregulated TEs compared to their overall genome proportion (chi‐square test, *p* < 0.001). This suggested that age‐dependent TE expression could contribute to TE mobilization; however, further experiments are needed to confirm this.

TEs can influence the expression of nearby genes through multiple mechanisms, including by modifying the epigenetic regulation of specific loci (Casacuberta and González 2013). We thus performed a permutation test to check whether DETEs found in older males were enriched nearby (±1 kb) differentially expressed genes (DEGs) (see Section 4). Overall, we found no differences (all *p* > 0.05) or statistically significant depletion (all *p* < 0.05) in both upregulated and downregulated TE insertions nearby genes (Table S14).

In summary, using a copy‐specific approach, comparisons between older male and older female flies revealed a greater number of TE copies with increased than decreased expression, a pattern that is already evident in young flies. The copy‐specific TE expression analysis revealed sex differences that were likely masked in family‐level analysis. Importantly, this pattern suggests that TE regulation in aging may operate at a local scale, with sex‐specific effects emerging at the level of individual TE copies rather than at the level of entire TE families. Such differences could arise from sex‐dependent chromatin environments, including the influence of Y‐linked heterochromatin acting in trans, or from differences in nuclear organization that modulate the accessibility and silencing of specific loci.

### 2.6 Increased TE Expression Is Not Consistently Associated With Reduced Male Lifespan

To investigate if the expression of some specific TE copies in older males compared to older females was associated with sex differences in lifespan, we performed longevity assays across all strains included in our transcriptome analysis.

Males showed reduced lifespan in both *D. melanogaster* and in one *D. simulans* strain (Log Rank test, all *p* < 0.001 except for dsgoth31, where *p* = 0.600). Moreover, negative SGL values were found in both species (Figures 4A–D, and Table S15). Nevertheless, no statistically significant differences in lifespan between males and females were observed in *D. suzukii* (Log Rank test, *p* = 0.400 and *p* = 0.500 for MT47 and S29, respectively) (Figure 4E,F and Table S15).

**FIGURE 4:**
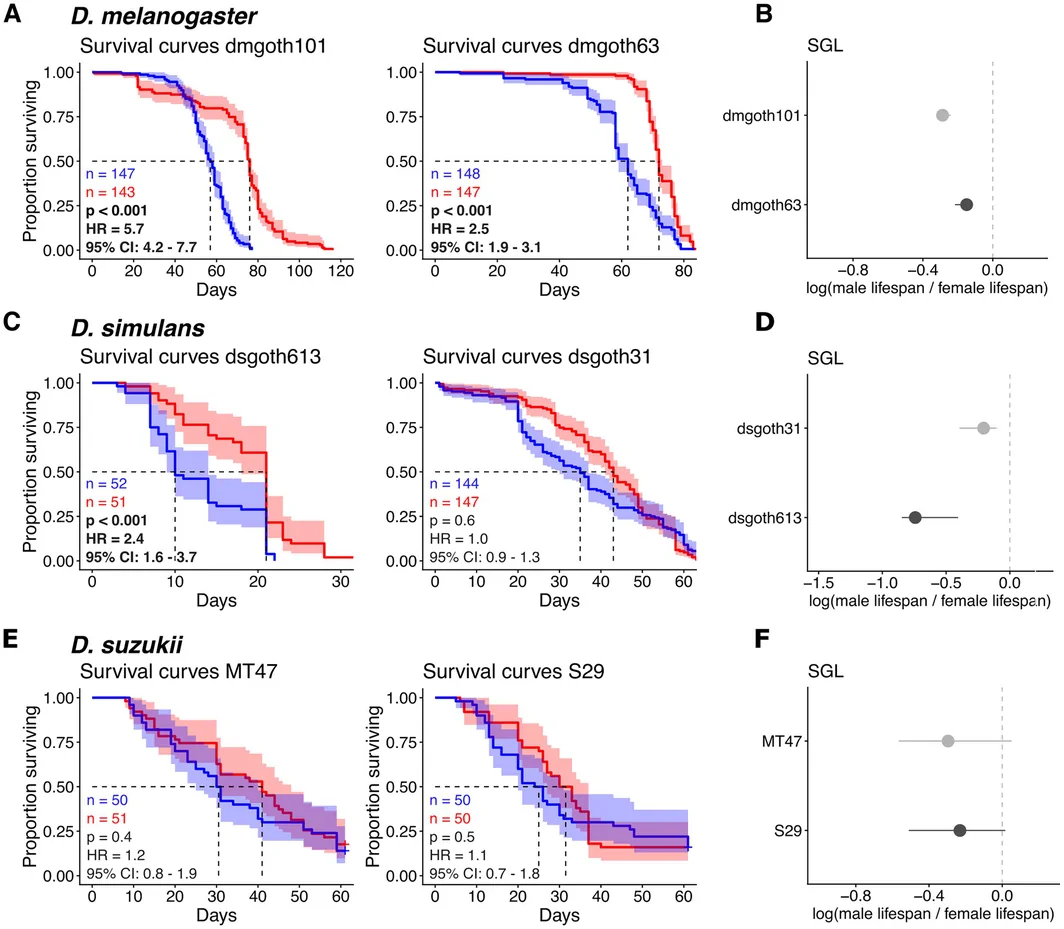
*D. melanogaster* and *D. simulans* males have reduced lifespan. Kaplan–Meier survivorship curves for males (blue) and females (red) in (A) *D. melanogaster* , (C) *D. simulans*, and (E) *D. suzukii* with the shaded region indicating the upper and lower 95% confidence interval calculated from the Kaplan–Meier curves. Dashed lines represent median lifespan values. Statistically significant *p* values and Cox proportional hazard analysis (HR) are highlighted in bold. SGL values measured as the natural logarithm of the ratio between male and female median lifespan are shown for each strain of (B) *D. melanogaster* , (D) *D. simulans*, and (F) *D. suzukii*. Dots represent mean SGL values and lower and upper whiskers indicate the upper and lower 95% confidence intervals.

While *D. melanogaster* strains showed reduced male lifespan and a higher number of DETEs in older males relative to older females (Figure 3B), these patterns were not consistently observed across species. Therefore, our data do not provide clear evidence for a general association between increased TE expression and reduced male lifespan. Consistent with this, when analyzing each strain individually, we found no correlation between male lifespan or SGL values and overall genomic TE content (Pearson correlation, all *p* > 0.05). Hence, TE content at the genome‐wide level does not appear to be directly linked to lifespan variations, as previously suggested for *D. melanogaster* and other species like the Greenland shark (Delanoue et al. 2023; Sahm et al. 2024).

### 2.7 Y Chromosome Replacement Is Associated to Increased SGL by Reducing Male Lifespan in *D. melanogaster*

To check whether the Y chromosome is associated with changes in SGL, we performed introgression experiments to replace the Y chromosome between different strains in both *D. melanogaster* and *D. simulans* (see Section 4).

In *D. melanogaster* , introgression crosses were made between dmgoth101 and dmsj7 strains, with longevity assays conducted over 10 generations (Figure 5A). dmgoth101 and dmsj7 were selected because they exhibited contrasting SGL values (−0.29, 95% CI [−0.32, −0.24] for dmgoth101; −0.07, 95% CI [−0.13, −0.04] for dmsj7) (Figure 5B). In addition, lifespan differed significantly between the two strains in both males and females (Log‐Rank test, *p* < 0.001 for each sex; hazard ratio = 0.24, 95% CI [0.18, 1.35] for males; hazard ratio = 0.58, 95% CI [0.46, 0.74] for females) (Figure 5C). These strains were also chosen because their genomes have been previously sequenced using long‐read technologies (Fablet et al. 2023).

**FIGURE 5:**
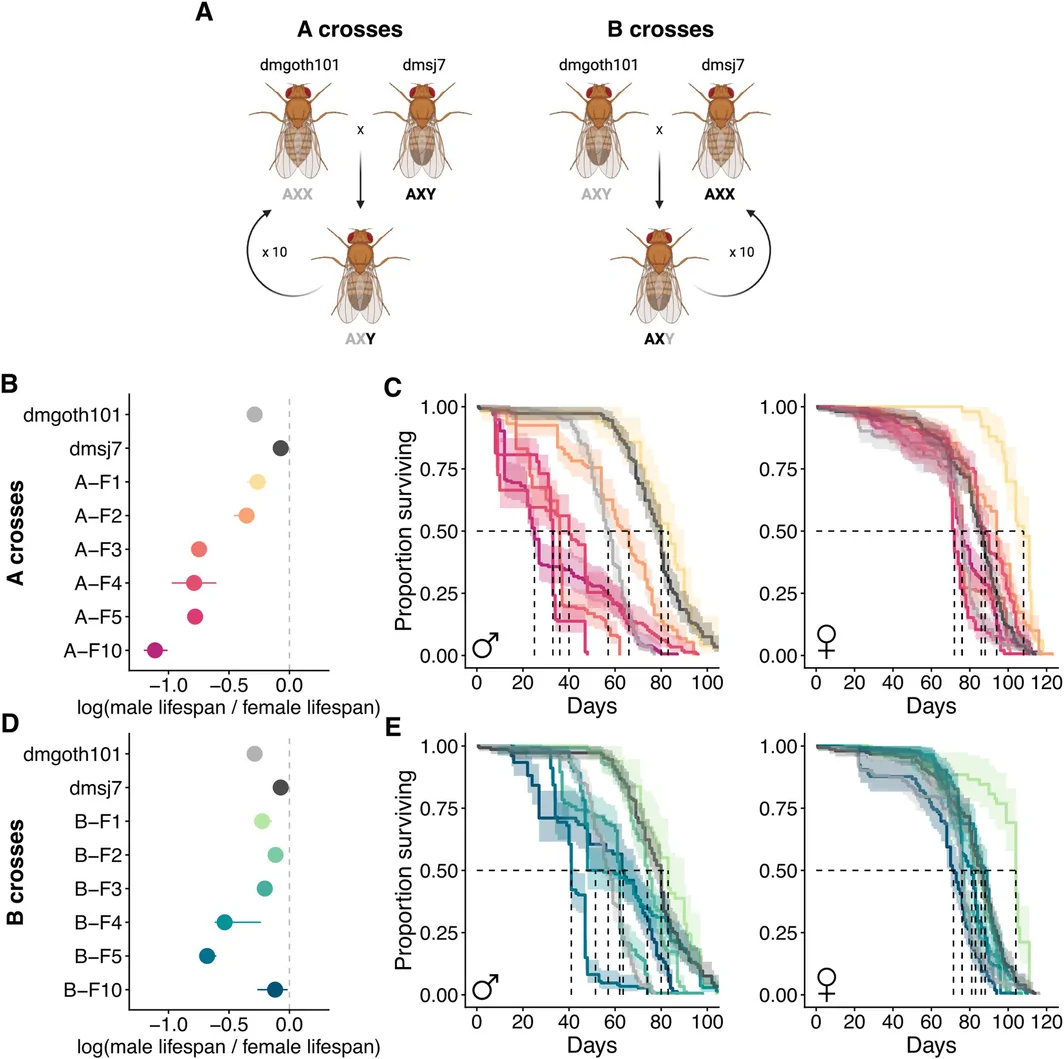
Y chromosome replacement is associated with reduced male lifespan in *D. melanogaster* . (A) Crossing scheme for both reciprocal crosses to obtain flies with Y chromosome replacements. “A” represents all autosomes. (B) SGL values measured as the natural logarithm of the ratio between male and female median lifespan for A reciprocal crosses. Results for maternal strains are shown in gray (dmgoth101) and black (dmsj7) while different colors represent generations of introgression from F1 to F10. Dots show mean SGL values and lower and upper whiskers indicate the upper and lower 95% confidence intervals. (C) Kaplan–Meier survivorship curves for males (left panel, *n* = 150 each generation) and females (right panel, *n* = 150 each generation) in A reciprocal crosses. Colors represent the different generations as in Panel B. Dashed lines represent median lifespan values. (D) SGL values measured as the natural logarithm of the ratio between male and female median lifespan for B reciprocal crosses. Results for maternal strains are shown in gray (dmgoth101) and black (dmsj7) while different colors represent generations of introgression from F1 to F10. Dots show mean SGL values and lower and upper whiskers indicate the upper and lower 95% confidence intervals. (E) Kaplan–Meier survivorship curves for males (left panel, *n* = 150 each generation) and females (right panel, *n* = 150 each generation) in B reciprocal crosses. Colors represent the different generations as in Panel D. Dashed lines represent median lifespan values.

We performed two types of crosses: “A crosses” started with dmgoth101 females and dmsj7 males, while “B crosses” were the reciprocal (Figure 5A). If the Y chromosome were solely responsible for the differences in SGL, we would expect reciprocal crosses to have opposite outcomes, with one showing a reduction and the other an increase in SGL, respectively. However, both crosses showed an increase in absolute SGL values across generations, primarily due to a reduction in male lifespan (Figure 5B,D and Table S16). In A crosses, SGL changed from −0.26 (95% CI [−0.35, −0.19]) to −1.11 (95% CI [−1.20, −1.01]) from generation F1 to F10 (Figure 5B). Similarly, B crosses showed an initial rise in SGL absolute values from −0.23 (95% CI [−0.29, −0.14]) to −0.68 (95% CI [−0.71, −0.60]) by F5, but decreased by F10 (95% CI [−0.27, −0.01]) (Figure 5D). In both reciprocal crosses, males exhibited a progressive reduction in lifespan across all generations, unlike females, whose lifespan showed less variation across generations (standard deviation of mean lifespan in A crosses: 22.21 for males and 12.13 for females; B crosses: 14.42 for males and 10.06 for females) (Figure 5C,E).

We then repeated the introgression crosses using again dmgoth101 and another strain, dmgoth63, which showed no major SGL differences compared to dmgoth101 (−0.15; 95% CI [−0.22, −0.13]) and in which neither males nor females exhibited statistically significant lifespan differences (Log‐Rank test, *p* = 0.669, hazard ratio = 1.07, 95% CI [0.85, 1.35]; and *p* = 0.686, hazard ratio = 1.87, 95% CI [1.46, 2.38], respectively) (Figure S12). This time, in both reciprocal crosses, introgression did not result in significant SGL value changes across generations (Figure S12 and Table S16). Furthermore, neither males nor females showed consistent changes in lifespan across generations (Figure S12). The mean lifespan remained stable, with low variability between generations or sexes (standard deviation of mean lifespan in A crosses: 4.54 for males and 3.95 for females; B crosses: 3.63 for males and 1.57 for females) (Figure S12).

Finally, we also performed introgression crosses between the *D. simulans* dsgoth31 and dssj27 strains with longevity assays conducted over five generations (Figure S13). Again, these strains were selected because they exhibited contrasting SGL values (−0.29, 95% CI [−0.32, −0.24] for dsgoth31; −0.07, 95% CI [−0.13, −0.04] for dssj27) and both have been previously sequenced using long‐read technologies (Figure S13) (Fablet et al. 2023). In this species, “A crosses” started with dsgoth31 females and dssj27 males, while “B crosses” were the reciprocal (Figure S13). Unlike in *D. melanogaster* , both reciprocal crosses showed no consistent pattern: SGL values fluctuated across generations rather than steadily increasing or decreasing (Figure S13). Moreover, lifespan also fluctuated, but with low variability between sexes (standard deviation of mean lifespan in A crosses: 14.19 for males and 13.78 for females; B crosses: 14.21 for males and 10.67 for females) (Figure S13).

In summary, we found that the replacement of the Y chromosome between strains with different SGL values was associated with a progressive increase in absolute SGL values due to a reduced male lifespan only in *D. melanogaster* . If SGL values were directly linked to the Y chromosome's TE content, we might expect the replacement of the Y chromosome to result in an SGL value matching that of the donor strain across generations. However, this was not observed. Instead, absolute SGL values increased in both reciprocal crosses when the Y chromosome was replaced between strains with different SGL values, suggesting an interaction between the Y chromosome and the genomic background, rather than a unique effect of Y‐linked TE content. In contrast, in *D. simulans* , we observed fluctuations in SGL values across generations, with similar standard deviations of mean lifespan in both sexes. These results highlight that the effects of Y chromosome replacement also differ between species.

### 2.8 Male Lifespan Is Not Associated With Y Chromosome Size

To exclude the possibility that variation in Y chromosome size is associated with SGL values, we estimated Y chromosome size indirectly using flow cytometry. This was achieved by comparing genome size between parental male flies and F1 male offspring from both reciprocal crosses in *D. melanogaster* and *D. simulans* (see Section 4, Figure S14, Table S17).

In *D. melanogaster* , we did not detect significant differences in genome size between dmgoth101 males and A cross F1 males (crosses between dmgoth101 females and dmsj7 males), nor between dmsj7 males and B cross F1 males (crosses between dmsj7 females and dmgoth101 males) (Pairwise Wilcoxon test, all *p* > 0.05) (Figure S14). These results suggested that the Y chromosomes of dmgoth101 and dmsj7 were similar in size and likely harbored comparable repeat content (Nguyen et al. 2022). Therefore, the previously observed changes in male lifespan across generations are unlikely to be driven by differences in Y chromosome size.

In *D. simulans* , we did observe significant differences in genome size between dsgoth31 males and F1 males from the A cross (crosses between dsgoth31 females and dssj27 males) (Pairwise Wilcoxon test, *p* = 0.017), with A cross F1 males exhibiting larger genome sizes (Figure S13). This observation suggested that the Y chromosome in dssj27 may be larger than that of dsgoth31. However, this pattern was not supported by the reciprocal cross: no significant differences in genome size were observed between dssj27 males and F1 males from the B cross (crosses between dssj27 females and dsgoth31 males) (Pairwise Wilcoxon test, *p* > 0.05), although B cross F1 males tended to have larger genome sizes (Figure S14). In this species, we did not detect a progressive reduction in male lifespan across generations, further suggesting that male lifespan is not associated with Y chromosome size.

### 2.9 Y Chromosome Replacement Is Associated With Global Gene Expression Changes and Increased TE Expression

In *D. melanogaster* , polymorphisms on the Y chromosome have been shown to modulate the expression of numerous X‐linked and autosomal genes with effects on fitness, temperature sensitivity of spermatogenesis, sex ratio distortion, and behavior (Carvalho et al. 1997; Chippindale and Rice 2001; Huttunen and Aspi 2003; Montchamp‐Moreau et al. 2001; Rohmer et al. 2004; Stoltenberg and Hirsch 1997). Since extremely low levels of DNA sequence polymorphism are observed in single‐copy Y‐linked genes, Y chromosome polymorphisms in the heterochromatin have been shown to serve as a major source of epigenetic variation interacting with chromatin components to modulate the expression of genome‐wide genes (Jiang et al. 2010; Lemos et al. 2008, 2010; Zurovcova and Eanes 1999).

To assess the impact of Y chromosome replacement on genome‐wide gene expression, we extracted RNA from male and female *D. melanogaster* flies at generations F1 and F10 from reciprocal introgression crosses between dmgoth101 and dmsj7 (see Section 4, Tables S18 and S19).

In agreement with the literature, F10 flies exhibited a substantial number of DEGs compared with F1 flies in both reciprocal crosses, with a greater number observed in males (A crosses: 919 DEGs in males and 232 in females; B crosses: 2031 DEGs in males and 1154 in females) (Figure 6A and Figure S15) (Jiang et al. 2010; Lemos et al. 2008, 2010). The gene ontology (GO) categories enriched in males are consistent with previous studies in *D. melanogaster* showing that Y chromosome replacement affects the expression of genes involved in transcriptional regulation, chromatin organization, DNA packaging, and metabolism (Figure S16) (Lemos et al. 2010).

**FIGURE 6:**
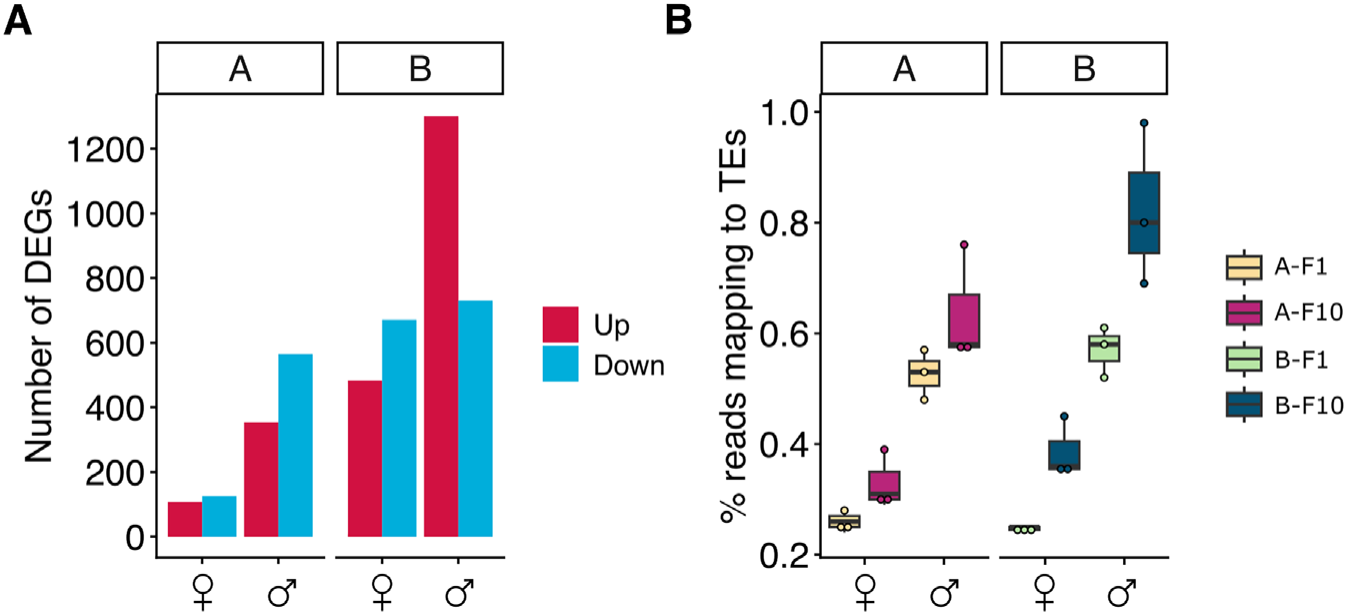
Y chromosome replacement is associated with global gene and TE expression changes in *D. melanogaster* . (A) Barplots showing the number of upregulated (in red) and downregulated (in blue) DEGs in F10 compared to F1 flies aging for both males and females of the different reciprocal crosses (A and B crosses). (B) Boxplots showing the percentage of reads mapping to TE sequences in the RNA‐seq data from F1 and F10 flies of both A and B reciprocal crosses. Boxplots show the median (horizontal line), first and third quartiles (lower and upper bounds, respectively), and minimum and maximum values (lower and upper whiskers, respectively). Each dot represents an independent biological replicate.

We then measured TE expression between F1 and F10 flies (Tables S18 and S19). We assessed the effects of reciprocal cross (A or B), generation (F1 or F10), sex, and their interactions on the percentage of TE‐derived reads (see Section 4). F10 flies had a higher percentage of TE‐derived reads than F1 flies in both sexes (GLM, estimate 0.105, *p* = 0.033), with differences more pronounced in B crosses (GLM, estimate 0.068, *p* = 0.048) (Figure 6B and Figure S15). In addition, males had more TE‐derived reads than females in both generations (GLM, estimate 0.295, *p* < 0.001), and the interaction between generation and sex was not statistically significant (GLM, estimate 0.077, *p* = 0.249) (Figure 6B). These findings suggest that the Y replacement might also be associated with an overall increase in TE transcripts across generations.

Altogether, we found that Y chromosome replacement between *D. melanogaster* strains dmgoth101 and dmsj7 was associated with global gene expression changes and with a general increase in TE expression in males compared to females already seen in the first introgression cross (F1). The percentage of TE‐derived reads increased in F10 compared to F1 in both sexes and reciprocal crosses.

## 3 Discussion

In this work, we characterized SGL variability across wild‐type populations in three *Drosophila* species, finding greater population‐level variance and an overall reduced male lifespan compared to females, consistent with previous studies (Yoon et al. 1990).

Sex differences in lifespan have been associated with TE expression in some organisms such as *D. melanogaster* and *D. miranda* (Brown et al. 2020b; Nguyen et al. 2022). Here, using a copy‐specific approach, we found a general age‐related increase in TE copies in both males and females of *D. melanogaster* and *D. suzukii*. This result suggests that there might be an age‐related derepression of TEs, likely due to a general loss of heterochromatin in repetitive regions in aged flies, as it was previously observed in *D. melanogaster* (Brown et al. 2020a, 2020b). Specifically, male flies from the same species have been shown to undergo a more rapid heterochromatin mark loss producing a more pronounced derepression of TEs in older males compared to older females (Brown et al. 2020a).

Previous studies in one *D. melanogaster* laboratory strain showed some differences in the total fraction of repetitive reads that increased during aging between males (4.2%) and females (2.6%) (Brown et al. 2020b). Here, we could not detect statistically significant effects of sex or the interaction between sex and age on the percentage of TE‐derived reads, indicating that age is associated with a similar increase in TE transcript levels in both males and females. In this work, the difference in fraction of TE‐derived reads between older and young samples was 1.47% in males and 1.37% in females from dmgoth63, and 0.07% in males and 0.01% in females from dsgoth31. Moreover, it was previously found in one laboratory strain that 32 TE families showed a significant increase in expression during aging in males while only six were found in females (Brown et al. 2020b). Although we did not follow the same approach, differential expression analysis between older males and older females did reveal more upregulated specific TE copies in all the species tested. The TE expression analysis at the copy level revealed sex differences that were likely diluted in family‐level assessments.

Particularly in *D. melanogaster* strains, in which there is a higher number of DETEs in older males relative to older females, we observed a reduced male lifespan. However, these patterns were not consistently observed across the other two *Drosophila* species. Therefore, our data do not provide clear evidence for a general association between increased TE expression and reduced male lifespan. Moreover, we found no association between SGL values and TE content in any of the three species tested. Indeed, if TE expression levels were directly linked to TE content, we would expect a stronger effect *in D. suzukii*. However, this was not observed: in this species there is only a weak tendency towards shorter lifespan in males.

Besides TE expression, it has been proposed that age might increase TE transposition, leading to a greater number of somatic TE insertions that impact genomic stability and cellular homeostasis, ultimately contributing to a decreased lifespan. In *D. melanogaster* , lifespan has been shown to decrease with increasing somatic genetic damage caused by *P element*, *mariner*, and *mdg4* insertions (Chang and Dubnau 2019; Li et al. 2013; Nikitin and Woodruff 1995; Wood et al. 2016; Woodruff and Nikitin 1995). Yet, some recent studies found that the number of new TE insertions does not seem to correlate with age, suggesting that TE expression instead could have a global impact in transcriptional dysfunction during aging and affecting lifespan (Pabis et al. 2024; Rigal et al. 2022; Treiber and Waddell 2017; Tsai et al. 2024; Yang et al. 2022).

This apparent discrepancy has been explored in long‐lived species, which often exhibit an overall reduction in both TE abundance and expression (Elsner et al. 2018; Ricci et al. 2023; Sturm et al. 2023; Teefy et al. 2023). However, exceptions exist where TEs might play a beneficial role in aging (Sahm et al. 2024; Sulak et al. 2016; Zhao et al. 2021). For instance, in blind mole rats ( *Spalax galili* ), TE activation can act as a tumor suppressor by triggering the innate immune system (Zhao et al. 2021). In the Greenland shark ( *Somniosus microcephalus* ), the longest‐lived vertebrate, TEs constitute 70.6% of the genome, the highest proportion described in sharks (Sahm et al. 2024). In this case, TE expansion may be linked to the expansion of DNA repair genes, which in turn may help to tolerate higher levels of TE activity (Sahm et al. 2024). Therefore, unraveling the complex mechanisms of delayed aging requires careful consideration of species‐specific features, including the abundance, composition, and distribution of TE in the genomes (Yushkova and Moskalev 2023).

Our results also revealed that, in *D. melanogaster*, the replacement of the Y chromosome between strains exhibiting differences in SGL values and male lifespan was associated with a progressive decline in male survival, leading to a more pronounced SGL across generations. Because both reciprocal crosses exhibited a similar reduction in male lifespan and parental strains had no significant differences in Y chromosome size estimations, we suggest that Y‐linked TE abundance alone cannot account for the observed effects. Similar experiments in *D. simulans* did not show a consistent decline in male lifespan across generations, but rather fluctuating patterns. Together, these results also suggest that the impact of the Y chromosome on longevity is species‐specific.

In agreement with our results, two recent works indicate that male survival is not linked to Y chromosome size (used as a proxy of different Y‐linked TE abundance) in both *D. melanogaster* and *D. pseudoobscura* (Delanoue et al. 2023; Nguyen et al. 2022). Hence, based on our introgression experiments, we hypothesize that interactions between the Y chromosome and the genetic background may offer a more plausible explanation for variation in male lifespan compared to Y chromosome sizes or TE abundance (Griffin et al. 2015; Jiang et al. 2010; Lemos et al. 2008, 2010). Indeed, it has been shown that *D. melanogaster* males differing only in their Y chromosomes showed a substantial number of DEGs, and this effect was more pronounced when males differed in their Y chromosome population origin (Jiang et al. 2010). Consistently, we also observed an impact of Y chromosome variation on genome‐wide transcription and TE expression changes. However, it is also conceivable that other genomic polymorphisms other than TEs on the Y chromosome are contributing to reduced lifespan, as well as mitochondrial genome–Y chromosome interactions (Ågren et al. 2020). Thus, while TE polymorphisms are strong candidates, other factors on the Y chromosome could also be linked to reduced lifespan.

In summary, our work shows that SGL is a variable trait in wild‐type *Drosophila* populations and suggests that the effects of the Y chromosome on male lifespan may be species‐specific. Moreover, our results prompt a reconsideration of the toxic Y hypothesis, as it may be less relevant in species with lower genomic TE content. Importantly, the effects of the Y chromosome on lifespan appear to be more closely associated with epistatic interactions with Y‐linked polymorphisms (especially in the heterochromatin) rather than overall TE abundance.

## 4 Methods

### 4.1 Fly Strains and Rearing

Samples of wild‐type populations of *D. melanogaster* , *D. simulans*, and *D. suzukii* were collected, and different isofemale strains were established for each species and geographical origin directly from gravid females collected in the field. A comprehensive list of all populations along with collection information is available in Table S1. *D. melanogaster* and *D. simulans* fly strains were reared in tubes with a nutritive “LM” medium containing agar, maize flour, yeast, Nipagine, ethanol, and water, while *D. suzukii* strains were reared in tubes with “Dalton” medium containing agar, cornmeal, yeast, sugar, Nipagine, ethanol, and water. Fly strains were kept in a 12:12 h light/dark cycle at 25°C.

### 4.2 Longevity Assays

#### 4.2.1 Longevity Assays for the Analysis of SGL Natural Variation in Different Drosophila Species

Fifty males that had emerged within the previous 8 h and 50 virgin females from each isofemale strain were placed in groups of 10 flies in tubes containing nutritive medium (“LM” for *D. melanogaster* and *D. simulans* and “Dalton” for *D. suzukii*). Each tube was considered as an independent biological replicate resulting in five replicates per sex and strain. Flies were separated by sex without anesthesia using an aspirator. Dead flies in every tube were counted every day, except for weekends, until 80% mortality was reached. Alive flies were placed in new tubes with fresh medium every week. Longevity assays were performed at 25°C and at a constant humidity of 60%.

#### 4.2.2 Longevity Assays in the Inbred Strains Included in the Transcriptome Analysis

For *D. melanogaster* strains, 150 males that had emerged within the previous 8 h and 150 virgin females from each strain were placed in groups of 10 flies in tubes containing “LM” medium. Each tube was considered as an independent biological replicate thus having 15 replicates per sex and strain. For *D. simulans* and *D. suzukii* strains, 50 males that had emerged within the previous 8 h and 50 virgin females from each strain were placed in groups of 10 flies in tubes containing nutritive medium (“LM” for *D. simulans* and “Dalton” for *D. suzukii*). Each tube was considered as an independent biological replicate resulting in five replicates per sex and strain. Dead flies in every tube were counted every day, except for weekends, until 80% mortality was reached. Alive flies were placed in new tubes with fresh medium every week. Longevity assays were performed at 25°C and at constant humidity of 60%.

#### 4.2.3 Longevity Assays in the Y Introgressed Strains

150 males that had emerged within the previous 8 h and 150 virgin females from each strain/generation of introgression were placed in groups of 10 flies in tubes containing “LM” medium. Again, each tube was considered as an independent biological replicate thus having 15 replicates per sex and strain/generation of introgression. Flies were separated by sex without anesthesia using an aspirator. Dead flies were counted every day, except for weekends, until all the flies in the tubes were dead. Alive flies were placed in new tubes with fresh medium every week. Longevity assays were performed at 25°C and at a constant humidity of 60%.

### 4.3 SGL Values Calculation

We estimated sex‐specific median adult lifespan (in days) for each strain/generation of introgression in our dataset (Tables S2, S15, and S16). The median adult lifespan corresponds to the time when 50% of the individuals in a particular strain are dead. To estimate the confidence interval for both males and females of each strain, we implemented a bootstrapping procedure. We performed 100,000 bootstrap iterations to calculate the median adult lifespan for each sample and then we calculated the natural logarithm (ln) of the ratio of median longevity between males and females (SGL values) (Lemaître, Ronget, and Gaillard 2020; Lemaître, Ronget, Tidière, et al. 2020). Confidence intervals for the difference in median longevity between males and females were computed using quantiles of the bootstrap of the logarithm ratios.

For the analysis of SGL natural variation, we used a Bayesian approach implemented in the *MCMCglmm* package in R to partition the variance and identify the contribution of species, population, and sampling variation to the observed differences in SGL. We fitted a hierarchical regression model including species as a fixed effect and population as a random effect, with standard error (SE) weighting for the longevity measurements. The priors were set as follows: for the residual variance, we used an inverse‐Wishart prior with nu = 0.002; for the population random effect, we used an inverse‐Wishart prior with nu = 1 and a large variance (*a* = 1000) for the prior mean (diff_median_longevity∼species + (1∣population) + idh(SE):units).

We also performed survival analysis using the Kaplan–Meier method and Cox proportional hazard models. The survival analysis was conducted separately for male and female flies within each strain/generation of introgression. We used the *survfit* function in R to estimate the survival probabilities over time based on the event status (dead or alive). Additionally, we generated survival curves using the *ggsurvplot* function from the *survminer* R package. These curves represent the estimated survival probabilities over time using a Kaplan–Meier estimation. Finally, we conducted pairwise comparisons to evaluate the significance of differences in survival distributions among various samples. This analysis was performed using the *pairwise_survdiff* function, applying the Benjamini–Hochberg correction method for multiple comparisons.

### 4.4 DNA Extraction, Nanopore Sequencing and Genome Assemblies

Genome assemblies for *D. melanogaster* dmgoth101 and dmgoth63 and for *D. simulans* strains dsgoth31 and dsgoth613 were obtained from Mohamed et al. (2020). To obtain genome assemblies of *D. suzukii*, we sequenced with Oxford Nanopore Technologies (ONT) two inbred strains: S29 and MT47. DNA was extracted using phenol chloroform from a pool of 10 adult females. The quantity and quality of genomic DNA were assessed using a NanoDrop One UV–Vis spectrophotometer (Thermo Fisher Scientific) and a Qubit 1.0 Fluorometer (Invitrogen). Three micrograms of DNA were then repaired with the NEBNext FFPE DNA Repair Mix (NEB M6630). End repair and dA‐tailing were carried out using the NEBNext End Repair/dA‐Tailing Module (E7546, NEB). DNA libraries were prepared using the Ligation Sequencing Kit 1D. MinION sequencing was conducted according to the manufacturer's instructions using R9.4.1 flow cells (FLO‐MIN106, ONT) and a Nanopore MinION Mk1b sequencer (ONT), operated by ONT MinKNOW (v.18.3.1). Following sequencing, base calling was performed using *Guppy* (v.4.0.5) in high‐accuracy mode.

Quality control of Nanopore reads was conducted using *NanoPlot* (v.1.10.2) (https://github.com/wdecoster/NanoPlot). Reads with a quality score below 7 were excluded from further analysis. Contig assembly was performed with *Flye* (v.2.8) (Kolmogorov et al. 2019) using default parameters, except for the *‐plasmids* option. All contigs were aligned with *minimap2* (v2.16) (H. Li 2018) using the ‐*x map‐ont* option, followed by four rounds of polishing with *RACON* (v1.3.2) (Vaser et al. 2017) under default settings. Assembly quality metrics were evaluated using *Assembly‐Stats* (v1.0.1) (https://github.com/sanger‐pathogens/assembly‐stats). Any assembly inconsistencies were manually reviewed with *D‐genies* (v1.2.0) (Cabanettes and Klopp 2018) and corrected using *samtools* (v1.9.0) with the *faidx* function, and *Gepard* (v1.4.0) was used to identify breaking points. Super scaffolding of all corrected assemblies was carried out with *RaGOO* (v.1.1), using the *‐s* and *‐t 4* parameters, based on the reference genome assembly (Paris et al. 2020). The resulting chromosome‐scale assemblies were then analyzed using Benchmarking Universal Single‐Copy Orthologs (BUSCO) (v.5.4.3) (Simão et al. 2015) with the *diptera_odb10* lineage (−l parameter). Gene annotation for the Nanopore genomes was completed with *Liftoff* (v.1.6.3), referencing the WT3_2.0 *D. suzukii* genome (Paris et al. 2020).

### 4.5 RNA‐Seq Extraction and Sequencing

RNA was extracted from six inbred strains, comprising two strains per species: *D. melanogaster* (dmgoth101 and dmgoth63), *D. simulans* (dsgoth31 and dsgoth613), and *D. suzukii* (MT47 and S29). For young samples, male and female flies of each strain were separately placed in several tubes with nutritive medium in groups of 20–25 flies. When flies were five days old, 30 flies per sex were dissected, and ovaries and testes were removed from female and male flies, respectively, and only carcasses were kept. For old samples, male and female flies of each strain were also separately placed in several tubes with nutritive medium in groups of 20–25 flies. 30 flies per sex were dissected, and ovaries and testes were removed from female and male flies, respectively, and only carcasses were kept. In total, three biological replicates per strain, age, and sex were performed. Ages at dissection and mortality percentages were as follows: dmgoth63 (males, 73 days, 42%; females, 51 days, 50%), dmgoth101 (males, 73 days, 50%; females, 38 days, 48%), dsgoth31 (males, 31 days, 63%; females, 20 days, 47%), dsgoth613 (males, 44 days, 46%; females, 31 days, 38%), MT47 (males, 21 days, 43%; females, 21 days, 38%), and S29 (males, 21 days, 18%; females, 21 days, 20%). Consequently, older samples may exhibit varying chronological ages across strains. Then, for both young and old samples, RNA was extracted from the obtained carcasses. For the Y chromosome replacement experiments, RNA was extracted from both F1 and F10 male and female flies from the two reciprocal crosses. Samples were obtained as mentioned before when flies were 5 days old.

RNA extraction was carried out using the RNeasy Mini kit (Qiagen) following the manufacturer's instructions and then a DNase treatment (Invitrogen) was performed. mRNA libraries were prepared with the Illumina TruSeq RNA Sample Prep Kit, following the manufacturer's guidelines. Libraries were sequenced on the Illumina HiSeq 4000 platform with paired‐end 100 nt reads and a minimum of 30 million reads per sample. The sequence raw files have been deposited in NCBI SRA under the BioProject accession number PRJNA1159534.

### 4.6 RNA‐Seq Mapping and Gene Quantification

Quality of the *fastq* sequencing files was assessed with FastQC (v.0.11.9). Then, *fastp* (v.0.23.4) was used to filter out bad reads, adapter removal and trimming (Chen 2023). Trimmed reads from *D. melanogaster* , *D. simulans*, and *D. suzukii* were then mapped against the Flybase *D. melanogaster* (r6.16) (Öztürk‐Çolak et al. 2024), the *D. simulans* (r2.02) (Öztürk‐Çolak et al. 2024), and *D. suzukii* WT3_2.0 (Paris et al. 2020) reference genomes, respectively with *STAR* (v.2.7.11a) (Dobin et al. 2013) using default parameters. On average, 92.7% of the reads were uniquely mapped to the corresponding genomes. To quantify gene expression, we used *htseq* (v.2.0.3) (Anders et al. 2015), with parameter *‐t exon*, which uses the sum of the reads aligned in all exons to assign counts for each gene.

### 4.7 TE Annotation

All the six strains used in the transcriptomic analysis (dmgoth101, dmgoth63, dsgoth31, dsgoth613, MT47, and S29) have been sequenced with long‐read sequencing technologies and genome assemblies were available or generated (Mérel et al. 2021; Mohamed et al. 2020). TE insertions were identified in the genome assemblies using *RepeatMasker* (v.4.1.2), which masks the genome where there are fragments homologous to consensus TE sequences. Parameters used were: *‐cutoff 250, −a, −s, −norna*. Consensus TE sequences used for *D. melanogaster* were obtained by using the parameter *‐species drosophila*. However, consensus sequences for *D. simulans* were obtained from https://github.com/bergmanlab/drosophila‐transposons/, while for *D. suzukii* from https://github.com/vmerel/DSu‐TE/tree/main/TE_db. Finally, *bed* files containing the coordinates of each annotated TE were created for every genome.

### 4.8 Differential Expression Analysis of Genes

Differential expression analyses of genes were conducted using the R package *DESeq2* (v.1.40.2) (Love et al. 2014). Raw read counts were merged into a unified matrix for each strain and subsequently normalized using default parameters (Tables S5–S7). Differential expression of genes was assessed between old and young samples in each strain separately. The log2 fold‐change was adjusted using the *apeglm* (v.1.22.1) algorithm from the *lfcshrink* function to minimize noise and preserve significant differences (Zhu et al. 2019). Genes were considered as significantly differentially expressed when the adjusted *p*‐value was < 0.05 and the absolute log2 fold‐change > 1.

### 4.9 Age‐Related Genes Found Differentially Expressed in Old Flies

Genes previously associated with aging in *D. melanogaster* were obtained from the Animal Aging and Longevity Database (AnAge) (https://genomics.senescence.info/genes/search.php?search.php?show=1&sort=1&page=1&organism=Drosophila%20melanogaster). From this list, only genes with a described “increased” lifespan effect were selected. Then, gene symbols were converted to FlyBase IDs with the *Batch Download* online tool (https://flybase.org/batchdownload). We finally looked for overlap of the age‐associated genes and the DEGs found in the young versus old samples comparisons. For *D. simulans* , we used the gene ortholog table available in FlyBase to look for correspondences in gene IDs. We could analyze a total of 149, 152, and 120 age‐associated genes in *D. melanogaster*, *D. simulans* , and *D. suzukii*, respectively.

### 4.10 Quantification and Differential Expression Analysis for TE Families

TE quantification at a family level was performed with *TEtools*, using the *TEcount* module (Lerat et al. 2017). One of the inputs of the *TEcount* module is the *rosette* file, a tabular file containing the names of each TE copy in the genome that easily groups them in TE families (or other criteria if needed) (Lerat et al. 2017). To do that, for each strain, we first build a *fasta* file with all the TE sequences annotated using *bedtools getfasta* and the previously generated *bed* file with TE annotations. Then, we generated the *rosette* file writing in the first column the name of the specific TE copy and in the second column the TE family in which it belongs. To assess whether the percentage of reads mapping to TE sequences varies with age, a GLM was fitted in R with Gamma error distribution and identity link function to test the effects of species, sex, age, and their interaction (sex × age) on TE‐derived reads (glm(alignment_rate~species + sex + age + sex:age, family = Gamma(link = “identity”))). The model showed a good fit (residual deviance = 7.0978, AIC = 42.136). Differential expression analysis of TE families was conducted using the R package *DESeq2* (v.1.40.2) as mentioned before, and merging in a single matrix gene and TE family counts (Tables S5–S7) (Love et al. 2014). TE families were considered as significantly differentially expressed when the adjusted *p*‐value was < 0.05 and the absolute log2 fold‐change > 1. TE families with a log2 fold change > 1 were classified as upregulated, whereas those with a log2 fold change < −1 were classified as downregulated. To assess whether the number of upregulated TE families was greater than the number of downregulated families in each sample, a one‐side binomial test was performed using the R function *binom.test* with the null hypothesis that up and downregulation were equally likely (*p* = 0.5). *p* values were calculated for each sample and subsequently adjusted for multiple testing using the Benjamini‐Hochberg procedure to control for false discovery rate.

### 4.11 RNA‐Seq Mapping, Quantification and Differential Expression Analysis for Specific TE Copies

TE copy‐specific expression was performed using *Telescope* (Bendall et al. 2019). Briefly, for each strain, trimmed paired‐end reads were aligned to the corresponding genome with *bowtie2* (v.2.5.1) using the parameter *‐k 100*. Then, again for each strain, we generated a *gtf* file containing all the TE copies present in the genome assembly following the format specified by *Telescope*. After that, the generated *sam* file was converted to *bam* with *samtools view* (v.1.17). Finally, for the locus‐specific quantification of TEs, the function *telescope assign* was used taking the already generated *gtf* and *bam* files as inputs. Differential expression analysis of TE copies was conducted using the R package *DESeq2* (v.1.40.2) as mentioned before, and merging in a single matrix gene and copy‐specific TE counts (Tables S8–S13) (Love et al. 2014). TE copies were considered as significantly differentially expressed when the adjusted *p*‐value was < 0.05 and the absolute log2 fold‐change > 1. TE copies with a log2 fold change > 1 were classified as upregulated, whereas those with a log2 fold change < −1 were classified as downregulated. To assess whether the number of upregulated DETEs families was greater than the number of downregulated ones in each sample, the same one‐side binomial test mentioned before was applied.

### 4.12 Identification of Full‐Length Insertions

Identification of full‐length insertion among the upregulated TE copies found between old males and females (adjusted *p*‐value was < 0.05 and absolute log2 fold‐change > 1) was performed with a generated *python* script that detects as full‐length copies the ones that are at least 80% of the length of the consensus sequence and with a Kimura divergence value < 20 (values obtained from the previously generated *RepeatMasker* output files). For *D. melanogaster* and *D. simulans* , TE consensus sequences were obtained from https://github.com/bergmanlab/drosophila‐transposons/, while *D. suzukii* consensus sequences were obtained from Mérel et al. (2021).

### 4.13 Permutation Tests

To test whether DETEs found in old male flies were enriched nearby DEGs (±1 kb), *bed* files were first generated with the ±1 kb flanking regions for these genes in each specific genome. *bed* files with the coordinates of all TE copies were also generated for each strain. Permutation tests were then performed using the R package *regioneR* (Gel et al. 2016). Specifically, TE positions were intersected with gene regions (±1 kb) using the *randomization = resampleRegions* parameter with 1000 permutations. Hence, for every up and downregulated gene set of size *n*, the number of genes containing at least one TE within the ±1 kb flanking regions was calculated. Under the null model, gene sets of size *n* were randomly sampled from all genes in the genome, and the number of genes with nearby TEs was recalculated for each permutation to generate a null distribution. *p* values were computed as the proportion of permutations in which the number of genes with nearby TEs was equal to or greater than the observed value (Table S14).

### 4.14 Y Chromosome Replacement Crosses

To check whether the Y chromosome is associated with changes in SGL, we performed introgression experiments to replace the Y chromosome between strains. The lack of recombination in *Drosophila* males enabled the generation of flies that differ only in the Y chromosome. Reciprocal crosses between the *D. melanogaster* strains dmgoth101 and dmsj7 were performed, between the dmgoth101 and dmgoth63, and between the *D. simulans* strains dsgoth31 and dssj27. Five virgin females and five males aged four to five days old were placed in a tube containing a nutritive medium, with five replicates performed for each reciprocal cross. Every two or three days, the flies were transferred to new tubes with fresh medium until four tubes were obtained for each reciprocal cross. This ensured enough offspring to carry out longevity assays and facilitated the continuation of introgression crosses. F1 male flies were subsequently crossed with the maternal strain, and the male offspring from each generation were consistently crossed with females from the maternal strain for five or 10 generations depending on the experiment. In each generation (except for generations F6–F9), longevity assays were carried out as previously described with 150 virgin females and 150 males from each of the five replicated reciprocal crosses (Table S16).

### 4.15 Genome Size Estimation

The genome size of *D. melanogaster* strains dmgoth101 and dmsj7, and *D. simulans* strains dsgoth31 and dssj27, as well as their intraspecific reciprocal cross offspring, was estimated separately for males and females using flow cytometry (Table S17). Nuclei were extracted from five heads of four days' old flies, using *D. virilis* as an internal control standard. Heads were homogenized in Galbraith buffer (30 mM trisodium citrate, 1:10.000 (v/v) triton X‐100, 2 μg/mL RNAse A, 20 mM MOPS, 21.3 mM MgCl_2_, pH 7.2) supplemented with 0.1 mg/mL propidium iodide. The homogenate was filtered twice through 140 and 30 μm nylon meshes. Samples were analyzed using a BD Accuri C6 flow cytometer equipped with a 488 nm argon laser. Relative fluorescence intensity was calculated by comparison with *D. virilis* , whose genome size is estimated at 0.34 pg (332 Mb) (Gregory and Johnston 2008). A total of 10 biological replicates were performed for each parental strain and for the intraspecific reciprocal cross offspring.

### 4.16 TE Transcript Quantification in Introgression Crosses

TE transcript quantification was performed with *TEtools*, using the *TEcount* module (Lerat et al. 2017). For each strain (dmgoth101 and dmsj7), a *fasta* file was first built with all the TE sequences annotated using *bedtools getfasta* and the previously generated *bed* file with TE annotations. Then, a *rosette* file per strain was generated writing in the first column the name of the specific TE copy and in the second column the TE family in which it belongs. After that, the two *rosette* files were merged to continue with the *TEtools* analysis. A GLM analysis was performed in R with Gaussian error distribution and identity link function test to assess the effects of the reciprocal cross, generation, sex, and the interaction between generation and sex on the percentage of TE‐derived reads (glm(alignment_rate~cross + generation + sex + generation:sex, family = gaussian (link = “identity”))). The model showed a good fit (residual deviance = 0.1188, AIC = −47.315).

### 4.17 Gene Ontology Enrichment Analysis

GO enrichment analysis was performed in R using the *clusterProfiler* package, comparing the F10 and F1 introgression generations from both A and B reciprocal crosses between dmgoth101 and dmsj7 strains (Xu et al. 2024). The list of DEGs previously obtained was used as input with gene identifiers provided in FlyBase format and the corresponding organism annotation database. The analysis considered all three GO ontologies (Biological Process, Molecular Function, and Cellular Component). The background gene set was defined as all genes tested in the experiment. Significantly enriched GO terms were identified using a *p*‐value and *q*‐value cutoff of 0.05, with multiple testing correction applied using the Benjamini‐Hochberg method. To reduce redundancy among enriched GO terms, results were simplified using the *simplify* function in *clusterProfiler*, applying a semantic similarity cutoff of 0.7 based on the Wang method and retaining the most significant term (lowest adjusted *p*‐value) within each cluster.

## Author Contributions

Conceived and designed the experiments: M.M., P.G., J.‐M.G., J.‐F.L., G.A.B.M., M.F., M.B., C.V. Performed the experiments: M.M., S.J., C.M.‐B., N.B., V.R.‐R., V.R. Analyzed the data: M.M., V.R. Contributed material/analysis tools: V.R. Wrote the paper: M.M.

## Funding

This work was supported by Agence Nationale de la Recherche, Projet‐ANR‐20‐CE02‐0015; HORIZON EUROPE Marie Sklodowska‐Curie Actions, 101065313; Fondation pour la Recherche Médicale.

## Conflicts of Interest

The authors declare no conflicts of interest.

## Supporting information


**Figure S1:** Natural variability in SGL in different *Drosophila* species. Histograms displaying the distribution of SGL values calculated as the logarithm of the ratio between male and female median lifespan. (A) Histogram displaying the distribution of SGL values per population in *D. melanogaster* , *D. simulans* , and *D. suzukii*. UK: United Kingdom, FR: France, FI: Finland, NL: the Netherlands, BR: Brazil, ES: Spain, RS: Serbia. (B) Histogram displaying the distribution of SGL values per species.
**Figure S2:** PCA analysis plot on normalized counts for genes (left), and TEs (right) in (A) *D. melanogaster* , (B) *D. simulans* , and (C) *D. suzukii*. PCA revealed that PC1 accounts for over 60% of the variance and separates samples by sex across all three Drosophila species. PC2 explained 7%–15% of the variance, distinguishing samples by age in *D. melanogaster* and *D. suzukii*, and by strain in *D. simulans* . PCA of TE counts revealed that in *D. melanogaster* , PC1 (43% variance) separates samples by age, while PC2 (21% variance) separates them by sex. In *D. simulans* and *D. suzukii*, PC1 explains 73% and 43% of the variance, respectively, separating samples by strain, while PC2 accounts for 10% and 24% of the variance, respectively, separating samples by age.
**Figure S3:** (A) Volcano plots of DEGs between old and young samples in all the species tested. Statistically significant upregulated genes are depicted in red and statistically significant downregulated genes are in blue. The name of the five most upregulated and downregulated genes in each strain are shown. (B) Barplots showing the number of upregulated (in red) and downregulated (in blue) genes between old and young samples in all the strains and species tested. Results for males are shown in upper panels while results for females are in lower panels.
**Figure S4:** Heatmaps showing the expression of previously‐associated aging genes in all the strains from *D. melanogaster* , *D. simulans* , and *D. suzukii*, averaged across replicates. Colors represent log2 fold changes, with red corresponding to upregulated genes and blue corresponding to downregulated genes. Significant *p* adjusted values are represented with asterisks.
**Figure S5:** Volcano plots of DE TE families between old and young samples in all the species tested. Statistically significant upregulated TE families are depicted in red and statistically significant downregulated TE families are in blue. The name of the five most upregulated and downregulated TE families in each strain are shown.
**Figure S6:** Upset plots showing the number of shared DE TE families between samples. Highlighted in red are the number of shared DE TE families between all the samples.
**Figure S7:** Volcano plots of DE TE families between old male flies and old female flies in all the species tested. Statistically significant upregulated TE families in old males are depicted in red and statistically significant downregulated TE families in old males are depicted in blue. The name of the five most upregulated and downregulated TE families in each strain are shown.
**Figure S8:** Volcano plots of DETE copies between old and young samples in all the species tested. Statistically significant upregulated TE copies are depicted in red and statistically significant downregulated TE copies are in blue.
**Figure S9:** Upset plots showing the number of shared DE TE copies between males and females from the same strain. Highlighted in red are the number of shared DE TE copies between both samples.
**Figure S10:** Volcano plots of DETE copies between old male flies and old female flies in all the species tested. Statistically significant upregulated TE copies are depicted in red and statistically significant downregulated TE copies are in blue.
**Figure S11:** Circular track plots showing the distribution of TE insertions (in gray) and upregulated TE copies (in red) in the genome of the *D. melanogaster* strains (plots represented in blue), *D. simulans* (orange), and *D. suzukii* (pink). Plots show the biggest scaffolds assembled for simplification. Circular track plots were generated with the *BioCircos* R package using for each strain its genome assembly and a *bed* file containing the coordinates of the annotated TEs and the upregulated TE copies in old males compared to old females.
**Figure S12:** (A) Crossing scheme for both reciprocal crosses to obtain flies with Y chromosome replacements. “A” represents all autosomes. (B) SGL values measured as the logarithm of the ratio between male and female median lifespan are shown for A reciprocal crosses. Results for maternal strains are shown in gray (dmgoth101) and black (dmgoth63) while different colors represent generations of introgression from F1 to F5. Dots show mean SGL values and lower and upper whiskers indicate the upper and lower 95% confidence intervals. (C) Kaplan–Meier survivorship curves for males (left panel, *n* = 150 each generation) and females (right panel, *n* = 150 each generation) in A reciprocal crosses. Colors represent the different generations as in Panel B. Dashed lines represent median lifespan values. (D) SGL values measured as the logarithm of the ratio between male and female median lifespan for B reciprocal crosses. Results for maternal strains are shown in gray (dmgoth101) and black (dmgoth63) while different colors represent generations of introgression from F1 to F5. Dots show mean SGL values and lower and upper whiskers indicate the upper and lower 95% confidence intervals. (E) Kaplan–Meier survivorship curves for males (left panel, *n* = 150 each generation) and females (right panel, *n* = 150 each generation) in B reciprocal crosses. Colors represent the different generations as in Panel D. Dashed lines represent median lifespan values.
**Figure S13:** (A) Crossing scheme for both reciprocal crosses to obtain flies with Y chromosome replacements. “A” represents all autosomes. (B) SGL values measured as the logarithm of the ratio between male and female median lifespan are shown for A reciprocal crosses. Results for maternal strains are shown in black (dsgoth31) and gray (dssj27) while different colors represent generations of introgression from F1 to F5. Dots show mean SGL values and lower and upper whiskers indicate the upper and lower 95% confidence intervals. (C) Kaplan–Meier survivorship curves for males (left panel, *n* = 150 each generation) and females (right panel, *n* = 150 each generation) in A reciprocal crosses. Colors represent the different generations as in Panel B. Dashed lines represent median lifespan values. (D) SGL values measured as the logarithm of the ratio between male and female median lifespan for B reciprocal crosses. Results for maternal strains are shown in black (dsgoth31) and gray (dssj27) while different colors represent generations of introgression from F1 to F5. Dots show mean SGL values and lower and upper whiskers indicate the upper and lower 95% confidence intervals. (E) Kaplan–Meier survivorship curves for males (left panel, *n* = 150 each generation) and females (right panel, *n* = 150 each generation) in B reciprocal crosses. Colors represent the different generations as in Panel D. Dashed lines represent median lifespan values.
**Figure S14:** (A) On the left, crossing scheme used to obtain male *D. melanogaster* flies for genome size estimation by flow cytometry. The picture illustrates reciprocal crosses between parental strains, indicating the origin of the Y chromosome and autosomal background in the resulting male progeny. On the right, genome size estimates obtained by flow cytometry for males from the parental strains and from the offspring of both reciprocal crosses (*n* = 50). Each point represents an individual measurement, and horizontal lines indicate the median. (B) The same as in (A), but for *D. simulans* . Asterisks indicate level of statistical significance of one‐sided Pairwise Wilcoxon tests (*p ≤ 0.05, **p ≤ 0.01, ***p ≤ 0.001).
**Figure S15:** (A) Scatter plots of genes comparing F10 and F1 introgression generations of both A and B reciprocal crosses between dmgoth101 and dmsj7 strains. Upregulated genes are shown in red while downregulated genes are shown in blue, under the cutoffs of FDR < 0.05 and log2 fold‐change > |1|. (B) Scatter plots of TE families comparing F10 and F1 introgression generations of both A and B reciprocal crosses between dmgoth101 and dmsj7 strains. Upregulated TE families are shown in red while downregulated TE families are shown in blue, under the cutoffs of FDR < 0.05 and log2 fold‐change > |1|.
**Figure S16:** GO analysis comparing F10 and F1 introgression generations of both A and B reciprocal crosses between dmgoth101 and dmsj7 strains.

**Table S1:** Information of the populations used.
**Table S2:** Longevity assays results for the analysis of SGL natural variation. SGL values were calculated as the logarithm of the ratio between male and female median lifespan. Low_CI and High_CI correspond to low and high 95% confidence intervals, respectively. SE corresponds to standard error. Statistically significant negative SGL values are highlighted in bold (females live longer), while statistically significant positive SGL values are highlighted in red (males live longer).
**Table S3:** List of upregulated genes, TE families and TE copies.
**Table S4:** List of downregulated genes, TE families and TE copies.
**Table S5:** Matrix of raw gene and TE family counts in *D. melanogaster* .
**Table S6:** Matrix of raw gene and TE family counts in *D. simulans* .
**Table S7:** Matrix of raw gene and TE family counts in D. suzukii.
**Table S8:** Matrix of raw TE copy counts in *D. melanogaster* dmgoth101. TE_ID specifies the chromosome, start position, end position, and TE family.
**Table S9:** Matrix of raw TE copy counts in *D. melanogaster* dmgoth63. TE_ID specifies the chromosome, start position, end position, and TE family.
**Table S10:** Matrix of raw TE copy counts in *D. simulans* dsgoth31. TE_ID specifies the chromosome, start position, end position, and TE family.
**Table S11:** Matrix of raw TE copy counts in *D. simulans* dsgoth613. TE_ID specifies the chromosome, start position, end position, and TE family.
**Table S12:** Matrix of raw TE copy counts in *D. suzukii* MT47. TE_ID specifies the chromosome, start position, end position, and TE family.
**Table S13:** Matrix of raw TE copy counts in *D. suzukii* S29. TE_ID specifies the chromosome, start position, end position, and TE family.
**Table S14:** *p*‐values from the permutation test performed with RegioneR. Significant *p*‐values highlighted in blue represent a significant depletion of DETEs nearby genes.
**Table S15:** Longevity assays results for the analysis of SGL natural variation. SGL values were calculated as the logarithm of the ratio between male and female mean lifespan. Low_CI and High_CI correspond to low and high 95% confidence intervals, respectively.
**Table S16:** Longevity assays results in the Y introgressed strains. SGL values were calculated as the logarithm of the ratio between male and female mean lifespan. Low_CI and High_CI correspond to low and high 95% confidence intervals, respectively.
**Table S17:** Flow citometry results.
**Table S18:** List of upregulated genes and TE families in introgression experiments ( *D. melanogaster* ).
**Table S19:** List of downregulated genes and TE families in introgression experiments ( *D. melanogaster* ).

## Data Availability

The sequencing data generated in this study has been submitted to the NCBI BioProject database (https://www.ncbi.nlm.nih.gov/bioproject/) under accession number PRJNA1159534. Any additional information required to reanalyze the data reported in this paper is available from the lead contact upon request.

## References

[acel70788-bib-0001] Ågren, J. A. , M. Munasinghe , and A. G. Clark . 2020. “Mitochondrial‐Y Chromosome Epistasis in *Drosophila melanogaster* .” Proceedings. Biological sciences 287, no. 1937: 20200469. 10.1098/rspb.2020.0469. PMC7661303 33081607

[acel70788-bib-0002] Anders, S. , P. T. Pyl , and W. Huber . 2015. “HTSeq—A Python Framework to Work With High‐Throughput Sequencing Data.” Bioinformatics 31, no. 2: 166–169. 10.1093/bioinformatics/btu638. PMC4287950 25260700

[acel70788-bib-0003] Bendall, M. L. , M. de Mulder , L. P. Iñiguez , et al. 2019. “Telescope: Characterization of the Retrotranscriptome by Accurate Estimation of Transposable Element Expression.” PLoS Computational Biology 15, no. 9: e1006453. 10.1371/journal.pcbi.1006453. PMC6786656 31568525

[acel70788-bib-0004] Bogu, G. K. , F. Reverter , M. A. Marti‐Renom , M. P. Snyder , and R. Guigó . 2019. “Atlas of Transcriptionally Active Transposable Elements in Human Adult Tissues.” (p. 714212). bioRxiv: The Preprint Server for Biology. 10.1101/714212.

[acel70788-bib-0005] Bourque, G. , K. H. Burns , M. Gehring , et al. 2018. “Ten Things You Should Know About Transposable Elements.” Genome Biology 19, no. 1: 199. 10.1186/s13059-018-1577-z. PMC6240941 30454069

[acel70788-bib-0006] Brengdahl, M. , C. M. Kimber , J. Maguire‐Baxter , and U. Friberg . 2018. “Sex Differences in Life Span: Females Homozygous for the X Chromosome Do Not Suffer the Shorter Life Span Predicted by the Unguarded X Hypothesis.” Evolution; International Journal of Organic Evolution 72, no. 3: 568–577. 10.1111/evo.13434. 29430636

[acel70788-bib-0092] Bronikowski, A. M. , R. P. Meisel , P. R. Biga , et al. 2022. “Sex‐Specific Aging in Animals: Perspective and Future Directions.” Aging Cell 21, no. 2: e13542. 10.1111/acel.13542. PMC8844111 35072344

[acel70788-bib-0007] Brooks, R. C. , and M. G. Garratt . 2017. “Life History Evolution, Reproduction, and the Origins of Sex‐Dependent Aging and Longevity.” Annals of the New York Academy of Sciences 1389, no. 1: 92–107. 10.1111/nyas.13302. 28009055

[acel70788-bib-0008] Brown, E. J. , A. H. Nguyen , and D. Bachtrog . 2020a. “The *Drosophila* Y Chromosome Affects Heterochromatin Integrity Genome‐Wide.” Molecular Biology and Evolution 37, no. 10: 2808–2824. 10.1093/molbev/msaa082. PMC7530609 32211857

[acel70788-bib-0009] Brown, E. J. , A. H. Nguyen , and D. Bachtrog . 2020b. “The Y Chromosome May Contribute to Sex‐Specific Ageing in *Drosophila* .” Nature Ecology & Evolution 4, no. 6: 853–862. 10.1038/s41559-020-1179-5. PMC7274899 32313175

[acel70788-bib-0010] Cabanettes, F. , and C. Klopp . 2018. “D‐GENIES: Dot Plot Large Genomes in an Interactive, Efficient and Simple Way.” PeerJ 6: e4958. 10.7717/peerj.4958. PMC5991294 29888139

[acel70788-bib-0011] Camus, M. F. , D. J. Clancy , and D. K. Dowling . 2012. “Mitochondria, Maternal Inheritance, and Male Aging.” Current Biology 22, no. 18: 1717–1721. 10.1016/j.cub.2012.07.018. 22863313

[acel70788-bib-0012] Carazo, P. , J. Green , I. Sepil , T. Pizzari , and S. Wigby . 2016. “Inbreeding Removes Sex Differences in Lifespan in a Population of *Drosophila melanogaster* .” Biology Letters 12, no. 6: 20160337. 10.1098/rsbl.2016.0337. PMC4938057 27354712

[acel70788-bib-0013] Carvalho, C. R. , B. A. Verdolin , and N. M. Vaz . 1997. “Indirect Effects of Oral Tolerance Cannot Be Ascribed to Bystander Suppression.” Scandinavian Journal of Immunology 45, no. 3: 276–281. 10.1046/j.1365-3083.1997.d01-394.x. 9122617

[acel70788-bib-0014] Casacuberta, E. , and J. González . 2013. “The Impact of Transposable Elements in Environmental Adaptation.” Molecular Ecology 22, no. 6: 1503–1517. 10.1111/mec.12170. 23293987

[acel70788-bib-0015] Cayuela, H. , J.‐M. Gaillard , C. Vieira , et al. 2023. “Sex Differences in Adult Lifespan and Aging Rate Across Mammals: A Test of the ‘Mother Curse Hypothesis’.” Mechanisms of Ageing and Development 212: 111799. 10.1016/j.mad.2023.111799. 36948470

[acel70788-bib-0016] Chang, Y.‐H. , and J. Dubnau . 2019. “The Gypsy Endogenous Retrovirus Drives Non‐Cell‐Autonomous Propagation in a *Drosophila* TDP‐43 Model of Neurodegeneration.” Current Biology 29, no. 19: 3135–3152. 10.1016/j.cub.2019.07.071. PMC6783360 31495585

[acel70788-bib-0017] Chen, H. , X. Zheng , D. Xiao , and Y. Zheng . 2016. “Age‐Associated De‐Repression of Retrotransposons in the *Drosophila* Fat Body, Its Potential Cause and Consequence.” Aging Cell 15, no. 3: 542–552. 10.1111/acel.12465. PMC4854910 27072046

[acel70788-bib-0018] Chen, S. 2023. “Ultrafast One‐Pass FASTQ Data Preprocessing, Quality Control, and Deduplication Using *Fastp* .” iMeta 2, no. 2: e107. 10.1002/imt2.107. PMC10989850 38868435

[acel70788-bib-0019] Chippindale, A. K. , and W. R. Rice . 2001. “Y Chromosome Polymorphism Is a Strong Determinant of Male Fitness in *Drosophila melanogaster* .” Proceedings of the National Academy of Sciences of the United States of America 98, no. 10: 5677–5682. 10.1073/pnas.101456898. PMC33272 11320221

[acel70788-bib-0020] Connallon, T. , I. J. Beasley , Y. McDonough , and F. Ruzicka . 2022. “How Much Does the Unguarded X Contribute to Sex Differences in Life Span?” Evolution Letters 6, no. 4: 319–329. 10.1002/evl3.292. PMC9346086 35937469

[acel70788-bib-0021] Copley, K. E. , and J. Shorter . 2023. “Repetitive Elements in Aging and Neurodegeneration.” Trends in Genetics: TIG 39, no. 5: 381–400. 10.1016/j.tig.2023.02.008. PMC10121923 36935218

[acel70788-bib-0022] De Cecco, M. , T. Ito , A. P. Petrashen , et al. 2019. “L1 Drives IFN in Senescent Cells and Promotes Age‐Associated Inflammation.” Nature 566, no. 7742: 73–78. 10.1038/s41586-018-0784-9. PMC6519963 30728521

[acel70788-bib-0023] Delanoue, R. , C. Clot , C. Leray , T. Pihl , and B. Hudry . 2023. “Y Chromosome Toxicity Does Not Contribute to Sex‐Specific Differences in Longevity.” Nature Ecology & Evolution 7, no. 8: 1245–1256. 10.1038/s41559-023-02089-7. PMC10406604 37308701

[acel70788-bib-0024] Dobin, A. , C. A. Davis , F. Schlesinger , et al. 2013. “STAR: Ultrafast Universal RNA‐Seq Aligner.” Bioinformatics 29, no. 1: 15–21. 10.1093/bioinformatics/bts635. PMC3530905 23104886

[acel70788-bib-0025] Driver, C. J. , and S. W. McKechnie . 1992. “Transposable Elements as a Factor in the Aging of *Drosophila melanogaster* .” Annals of the New York Academy of Sciences 673: 83–91. 10.1111/j.1749-6632.1992.tb27439.x. 1336649

[acel70788-bib-0026] Elsner, D. , K. Meusemann , and J. Korb . 2018. “Longevity and Transposon Defense, the Case of Termite Reproductives.” Proceedings of the National Academy of Sciences of the United States of America 115, no. 21: 5504–5509. 10.1073/pnas.1804046115. PMC6003524 29735660

[acel70788-bib-0027] Erlandsson, R. , J. F. Wilson , and S. Pääbo . 2000. “Sex Chromosomal Transposable Element Accumulation and Male‐Driven Substitutional Evolution in Humans.” Molecular Biology and Evolution 17, no. 5: 804–812. 10.1093/oxfordjournals.molbev.a026359. 10779541

[acel70788-bib-0028] Fabian, D. K. , H. M. Dönertaş , M. Fuentealba , L. Partridge , and J. M. Thornton . 2021. “Transposable Element Landscape in Drosophila Populations Selected for Longevity.” Genome Biology and Evolution 13, no. 4: evab031. 10.1093/gbe/evab031. PMC8355499 33595657

[acel70788-bib-0029] Fablet, M. , J. Salces‐Ortiz , A. Jacquet , et al. 2023. “A Quantitative, Genome‐Wide Analysis in Drosophila Reveals Transposable Elements’ Influence on Gene Expression Is Species‐Specific.” Genome Biology and Evolution 15, no. 9: evad160. 10.1093/gbe/evad160. PMC10492446 37652057

[acel70788-bib-0030] Frank, S. A. , and L. D. Hurst . 1996. “Mitochondria and Male Disease.” Nature 383, no. 6597: 224. 10.1038/383224a0. 8805695

[acel70788-bib-0031] Gatti, M. , and S. Pimpinelli . 1992. “Functional Elements in *Drosophila melanogaster* Heterochromatin.” Annual Review of Genetics 26: 239–275. 10.1146/annurev.ge.26.120192.001323. 1482113

[acel70788-bib-0032] Gel, B. , A. Díez‐Villanueva , E. Serra , M. Buschbeck , M. A. Peinado , and R. Malinverni . 2016. “regioneR: An R/Bioconductor Package for the Association Analysis of Genomic Regions Based on Permutation Tests.” Bioinformatics 32, no. 2: 289–291. 10.1093/bioinformatics/btv562. PMC4708104 26424858

[acel70788-bib-0033] Gregory, T. R. , and J. S. Johnston . 2008. “Genome Size Diversity in the Family Drosophilidae.” Heredity 101, no. 3: 228–238. 10.1038/hdy.2008.49. 18523443

[acel70788-bib-0034] Griffin, R. M. , D. Le Gall , H. Schielzeth , and U. Friberg . 2015. “Within‐Population Y‐Linked Genetic Variation for Lifespan in *Drosophila melanogaster* .” Journal of Evolutionary Biology 28, no. 11: 1940–1947. 10.1111/jeb.12708. 26230387

[acel70788-bib-0035] Huttunen, S. , and J. Aspi . 2003. “Complex Inheritance of Male Courtship Song Characters in *Drosophila virilis* .” Behavior Genetics 33, no. 1: 17–24. 10.1023/a:1021095331850. 12645818

[acel70788-bib-0036] Jiang, P.‐P. , D. L. Hartl , and B. Lemos . 2010. “Y Not a Dead End: Epistatic Interactions Between Y‐Linked Regulatory Polymorphisms and Genetic Background Affect Global Gene Expression in *Drosophila melanogaster* .” Genetics 186, no. 1: 109–118. 10.1534/genetics.110.118109. PMC2940279 20551438

[acel70788-bib-0037] Kolmogorov, M. , J. Yuan , Y. Lin , and P. A. Pevzner . 2019. “Assembly of Long, Error‐Prone Reads Using Repeat Graphs.” Nature Biotechnology 37, no. 5: 540–546. 10.1038/s41587-019-0072-8. 30936562

[acel70788-bib-0038] Lemaître, J.‐F. , V. Ronget , and J.‐M. Gaillard . 2020. “Female Reproductive Senescence Across Mammals: A High Diversity of Patterns Modulated by Life History and Mating Traits.” Mechanisms of Ageing and Development 192: 111377. 10.1016/j.mad.2020.111377. 33031803

[acel70788-bib-0039] Lemaître, J.‐F. , V. Ronget , M. Tidière , et al. 2020. “Sex Differences in Adult Lifespan and Aging Rates of Mortality Across Wild Mammals.” Proceedings of the National Academy of Sciences of the United States of America 117, no. 15: 8546–8553. 10.1073/pnas.1911999117. PMC7165438 32205429

[acel70788-bib-0040] Lemos, B. , L. O. Araripe , and D. L. Hartl . 2008. “Polymorphic Y Chromosomes Harbor Cryptic Variation With Manifold Functional Consequences.” Science 319, no. 5859: 91–93. 10.1126/science.1148861. 18174442

[acel70788-bib-0041] Lemos, B. , A. T. Branco , and D. L. Hartl . 2010. “Epigenetic Effects of Polymorphic Y Chromosomes Modulate Chromatin Components, Immune Response, and Sexual Conflict.” Proceedings of the National Academy of Sciences of the United States of America 107, no. 36: 15826–15831. 10.1073/pnas.1010383107. PMC2936610 20798037

[acel70788-bib-0042] Lerat, E. , M. Fablet , L. Modolo , H. Lopez‐Maestre , and C. Vieira . 2017. “TEtools Facilitates Big Data Expression Analysis of Transposable Elements and Reveals an Antagonism Between Their Activity and That of piRNA Genes.” Nucleic Acids Research 45, no. 4: e17. 10.1093/nar/gkw953. PMC5389681 28204592

[acel70788-bib-0043] Li, H. 2018. “Minimap2: Pairwise Alignment for Nucleotide Sequences.” Bioinformatics 34, no. 18: 3094–3100. 10.1093/bioinformatics/bty191. PMC6137996 29750242

[acel70788-bib-0044] Li, W. , L. Prazak , N. Chatterjee , et al. 2013. “Activation of Transposable Elements During Aging and Neuronal Decline in *Drosophila* .” Nature Neuroscience 16, no. 5: 529–531. 10.1038/nn.3368. PMC3821974 23563579

[acel70788-bib-0045] Love, M. I. , W. Huber , and S. Anders . 2014. “Moderated Estimation of Fold Change and Dispersion for RNA‐Seq Data With DESeq2.” Genome Biology 15, no. 12: 550. 10.1186/s13059-014-0550-8. PMC4302049 25516281

[acel70788-bib-0046] Maklakov, A. A. , S. J. Simpson , F. Zajitschek , et al. 2008. “Sex‐Specific Fitness Effects of Nutrient Intake on Reproduction and Lifespan.” Current Biology 18, no. 14: 1062–1066. 10.1016/j.cub.2008.06.059. 18635354

[acel70788-bib-0047] Marais, G. A. B. , J.‐M. Gaillard , C. Vieira , et al. 2018. “Sex Gap in Aging and Longevity: Can Sex Chromosomes Play a Role?” Biology of Sex Differences 9, no. 1: 33. 10.1186/s13293-018-0181-y. PMC6050741 30016998

[acel70788-bib-0048] Mérel, V. , M. Boulesteix , M. Fablet , and C. Vieira . 2020. “Transposable Elements in *Drosophila* .” Mobile DNA 11: 23. 10.1186/s13100-020-00213-z. PMC7334843 32636946

[acel70788-bib-0049] Mérel, V. , P. Gibert , I. Buch , et al. 2021. “The Worldwide Invasion of *Drosophila suzukii* Is Accompanied by a Large Increase of Transposable Element Load and a Small Number of Putatively Adaptive Insertions.” Molecular Biology and Evolution 38, no. 10: 4252–4267. 10.1093/molbev/msab155. PMC8476158 34021759

[acel70788-bib-0050] Merenciano, M. , A. Larue , G. Cristian , V. Cristina , R. Rita , and G. Clément . 2024. “Epigenetics and Genotypic Variation: A Transposable Elements’ Perspective.” In On Epigenetics and Evolution, 119–151. Academic Press. 10.1016/B978-0-443-19051-3.00006-1.

[acel70788-bib-0051] Merenciano, M. , A. Larue , C. Garambois , W. V. B. Nunes , and C. Vieira . 2025. “Exploring the Relationship of Transposable Elements and Ageing: Causes and Consequences.” Genome Biology and Evolution 17, no. 6: evaf088. 10.1093/gbe/evaf088. PMC12127037 40373205

[acel70788-bib-0052] Mohamed, M. , N. T.‐M. Dang , Y. Ogyama , et al. 2020. “A Transposon Story: From TE Content to TE Dynamic Invasion of *Drosophila* Genomes Using the Single‐Molecule Sequencing Technology From Oxford Nanopore.” Cells 9, no. 8: 1776. 10.3390/cells9081776. PMC7465170 32722451

[acel70788-bib-0053] Montchamp‐Moreau, C. , V. Ginhoux , and A. Atlan . 2001. “The Y Chromosomes of *Drosophila simulan*s Are Highly Polymorphic for Their Ability to Suppress Sex‐Ratio Drive.” Evolution; International Journal of Organic Evolution 55, no. 4: 728–737. 10.1554/0014-3820(2001)055[0728:tycods]2.0.co;2. 11392391

[acel70788-bib-0054] Narayan, V. P. , A. J. Wilson , and S. F. Chenoweth . 2022. “Genetic and Social Contributions to Sex Differences in Lifespan in Drosophila Serrata.” Journal of Evolutionary Biology 35, no. 4: 657–663. 10.1111/jeb.13992. PMC9314142 35290690

[acel70788-bib-0055] Nguyen, A. H. , and D. Bachtrog . 2021. “Toxic Y Chromosome: Increased Repeat Expression and Age‐Associated Heterochromatin Loss in Male *Drosophila* With a Young Y Chromosome.” PLoS Genetics 17, no. 4: e1009438. 10.1371/journal.pgen.1009438. PMC8061872 33886541

[acel70788-bib-0056] Nguyen, A. H. , W. Wang , E. Chong , K. Chatla , and D. Bachtrog . 2022. “Transposable Element Accumulation Drives Size Differences Among Polymorphic Y Chromosomes in *Drosophila* .” Genome Research 32, no. 6: 1074–1088. 10.1101/gr.275996.121. PMC9248874 35501131

[acel70788-bib-0057] Nikitin, A. G. , and R. C. Woodruff . 1995. “Somatic Movement of the Mariner Transposable Element and Lifespan of *Drosophila* Species.” Mutation Research 338, no. 1–6: 43–49. 10.1016/0921-8734(95)00010-4. 7565881

[acel70788-bib-0058] Öztürk‐Çolak, A. , S. J. Marygold , G. Antonazzo , et al. 2024. “FlyBase: Updates to the *Drosophila* Genes and Genomes Database.” Genetics 227, no. 1: iyad211. 10.1093/genetics/iyad211. PMC11075543 38301657

[acel70788-bib-0059] Pabis, K. , D. Barardo , O. Sirbu , K. Selvarajoo , J. Gruber , and B. K. Kennedy . 2024. “A Concerted Increase in Readthrough and Intron Retention Drives Transposon Expression During Aging and Senescence.” eLife 12: RP87811. 10.7554/eLife.87811. PMC10990488 38567944

[acel70788-bib-0060] Paris, M. , R. Boyer , R. Jaenichen , et al. 2020. “Near‐Chromosome Level Genome Assembly of the Fruit Pest *Drosophila suzukii* Using Long‐Read Sequencing.” Scientific Reports 10, no. 1: 11227. 10.1038/s41598-020-67373-z. PMC7343843 32641717

[acel70788-bib-0061] Peona, V. , O. M. Palacios‐Gimenez , J. Blommaert , et al. 2021. “The Avian W Chromosome Is a Refugium for Endogenous Retroviruses With Likely Effects on Female‐Biased Mutational Load and Genetic Incompatibilities.” Philosophical Transactions of the Royal Society of London. Series B, Biological Sciences 376, no. 1833: 20200186. 10.1098/rstb.2020.0186. PMC8310711 34304594

[acel70788-bib-0062] Pipoly, I. , V. Bókony , M. Kirkpatrick , P. F. Donald , T. Székely , and A. Liker . 2015. “The Genetic Sex‐Determination System Predicts Adult Sex Ratios in Tetrapods.” Nature 527, no. 7576: 91–94. 10.1038/nature15380. 26444239

[acel70788-bib-0063] Post, F. , E. Bornberg‐Bauer , M. Vasseur‐Cognet , and M. C. Harrison . 2023. “More Effective Transposon Regulation in Fertile, Long‐Lived Termite Queens Than in Sterile Workers.” Molecular Ecology 32, no. 2: 369–380. 10.1111/mec.16753. 36320186

[acel70788-bib-0064] Ricci, M. , V. Peona , A. Boattini , and C. Taccioli . 2023. “‘Comparative Analysis of Bats and Rodents’ Genomes Suggests a Relation Between Non‐LTR Retrotransposons, Cancer Incidence, and Ageing.” Scientific Reports 13, no. 1: 9039. 10.1038/s41598-023-36006-6. PMC10239488 37270634

[acel70788-bib-0065] Rigal, J. , A. Martin Anduaga , E. Bitman , E. Rivellese , S. Kadener , and M. T. Marr . 2022. “Artificially Stimulating Retrotransposon Activity Increases Mortality and Accelerates a Subset of Aging Phenotypes in *Drosophila* .” eLife 11: e80169. 10.7554/eLife.80169. PMC9427105 35980024

[acel70788-bib-0066] Rohmer, C. , J. R. David , B. Moreteau , and D. Joly . 2004. “Heat Induced Male Sterility in *Drosophila melanogaster*: Adaptive Genetic Variations Among Geographic Populations and Role of the Y Chromosome.” Journal of Experimental Biology 207, no. Pt 16: 2735–2743. 10.1242/jeb.01087. 15235002

[acel70788-bib-0067] Sahm, A. , A. Cherkasov , H. Liu , et al. 2024. “The Greenland Shark (*Somniosus microcephalus*) Genome Provides Insights Into Extreme Longevity.” (p. 2024.09.09.611499). bioRxiv: The Preprint Server for Biology. 10.1101/2024.09.09.611499.

[acel70788-bib-0068] Simão, F. A. , R. M. Waterhouse , P. Ioannidis , E. V. Kriventseva , and E. M. Zdobnov . 2015. “BUSCO: Assessing Genome Assembly and Annotation Completeness With Single‐Copy Orthologs.” Bioinformatics 31, no. 19: 3210–3212. 10.1093/bioinformatics/btv351. 26059717

[acel70788-bib-0069] Slotkin, R. K. , and R. Martienssen . 2007. “Transposable Elements and the Epigenetic Regulation of the Genome.” Nature Reviews. Genetics 8, no. 4: 272–285. 10.1038/nrg2072. 17363976

[acel70788-bib-0070] Stoltenberg, S. F. , and J. Hirsch . 1997. “Y‐Chromosome Effects on *Drosophila* Geotaxis Interact With Genetic or Cytoplasmic Background.” Animal Behaviour 53, no. Pt 4: 853–864. 10.1006/anbe.1996.0351. 11540408

[acel70788-bib-0071] Sturm, Á. , É. Saskői , B. Hotzi , et al. 2023. “Downregulation of Transposable Elements Extends Lifespan in *Caenorhabditis elegans* .” Nature Communications 14, no. 1: 5278. 10.1038/s41467-023-40957-9. PMC10465613 37644049

[acel70788-bib-0072] Sulak, M. , L. Fong , K. Mika , et al. 2016. “TP53 Copy Number Expansion Is Associated With the Evolution of Increased Body Size and an Enhanced DNA Damage Response in Elephants.” eLife 5: e11994. 10.7554/eLife.11994. PMC5061548 27642012

[acel70788-bib-0073] Teefy, B. B. , A. Adler , A. Xu , K. Hsu , P. P. Singh , and B. A. Benayoun . 2023. “Dynamic Regulation of Gonadal Transposon Control Across the Lifespan of the Naturally Short‐Lived African Turquoise Killifish.” Genome Research 33, no. 1: 141–153. 10.1101/gr.277301.122. PMC9977155 36577520

[acel70788-bib-0074] Teoli, J. , M. Merenciano , M. Fablet , et al. 2025. “Transposable Element Expression Is Associated With Sex Chromosome Number in Humans.” PLoS Genetics 21, no. 6: e1011668. 10.1371/journal.pgen.1011668. PMC12225981 40570058

[acel70788-bib-0075] Treiber, C. D. , and S. Waddell . 2017. “Resolving the Prevalence of Somatic Transposition in *Drosophila* .” eLife 6: e28297. 10.7554/eLife.28297. PMC5553932 28742021

[acel70788-bib-0076] Tsai, Y.‐T. , N. Seymen , I. R. Thompson , et al. 2024. “Expression of Most Retrotransposons in Human Blood Correlates With Biological Aging.” eLife 13: RP96575. 10.7554/eLife.96575. PMC11486490 39417397

[acel70788-bib-0077] Van Meter, M. , M. Kashyap , S. Rezazadeh , et al. 2014. “SIRT6 Represses LINE1 Retrotransposons by Ribosylating KAP1 but This Repression Fails With Stress and Age.” Nature Communications 5: 5011. 10.1038/ncomms6011. PMC4185372 25247314

[acel70788-bib-0078] Vaser, R. , I. Sović , N. Nagarajan , and M. Šikić . 2017. “Fast and Accurate De Novo Genome Assembly From Long Uncorrected Reads.” Genome Research 27, no. 5: 737–746. 10.1101/gr.214270.116. PMC5411768 28100585

[acel70788-bib-0079] Vinogradov, A. E. 1998. “Male Reproductive Strategy and Decreased Longevity.” Acta Biotheoretica 46, no. 2: 157–160. 10.1023/a:1001181921303. 9691260

[acel70788-bib-0080] Warmuth, V. M. , M. H. Weissensteiner , and J. B. W. Wolf . 2022. “Accumulation and Ineffective Silencing of Transposable Elements on an Avian W Chromosome.” Genome Research 32, no. 4: 671–681. 10.1101/gr.275465.121. PMC8997356 35149543

[acel70788-bib-0081] Wood, J. G. , and S. L. Helfand . 2013. “Chromatin Structure and Transposable Elements in Organismal Aging.” Frontiers in Genetics 4: 274. 10.3389/fgene.2013.00274. PMC3849598 24363663

[acel70788-bib-0082] Wood, J. G. , B. C. Jones , N. Jiang , et al. 2016. “Chromatin‐Modifying Genetic Interventions Suppress Age‐Associated Transposable Element Activation and Extend Life Span in *Drosophila* .” Proceedings of the National Academy of Sciences of the United States of America 113, no. 40: 11277–11282. 10.1073/pnas.1604621113. PMC5056045 27621458

[acel70788-bib-0083] Woodruff, R. C. , and A. G. Nikitin . 1995. “P DNA Element Movement in Somatic Cells Reduces Lifespan in *Drosophila melanogaster*: Evidence in Support of the Somatic Mutation Theory of Aging.” Mutation Research 338, no. 1–6: 35–42. 10.1016/0921-8734(95)00009-u. 7565880

[acel70788-bib-0084] Xirocostas, Z. A. , S. E. Everingham , and A. T. Moles . 2020. “The Sex With the Reduced Sex Chromosome Dies Earlier: A Comparison Across the Tree of Life.” Biology Letters 16, no. 3: 20190867. 10.1098/rsbl.2019.0867. PMC7115182 32126186

[acel70788-bib-0085] Xu, S. , E. Hu , Y. Cai , et al. 2024. “Using ClusterProfiler to Characterize Multiomics Data.” Nature Protocols 19, no. 11: 3292–3320. 10.1038/s41596-024-01020-z. 39019974

[acel70788-bib-0086] Yang, N. , S. P. Srivastav , R. Rahman , et al. 2022. “Transposable Element Landscapes in Aging *Drosophila* .” PLoS Genetics 18, no. 3: e1010024. 10.1371/journal.pgen.1010024. PMC8893327 35239675

[acel70788-bib-0087] Yoon, J. S. , K. P. Gagen , and D. L. Zhu . 1990. “Longevity of 68 Species of *Drosophila* .” Ohio Journal of Science 90: 16–32.

[acel70788-bib-0088] Yushkova, E. , and A. Moskalev . 2023. “Transposable Elements and Their Role in Aging.” Ageing Research Reviews 86: 101881. 10.1016/j.arr.2023.101881. 36773759

[acel70788-bib-0089] Zhao, Y. , E. Oreskovic , Q. Zhang , et al. 2021. “Transposon‐Triggered Innate Immune Response Confers Cancer Resistance to the Blind Mole Rat.” Nature Immunology 22, no. 10: 1219–1230. 10.1038/s41590-021-01027-8. PMC8488014 34556881

[acel70788-bib-0090] Zhu, A. , J. G. Ibrahim , and M. I. Love . 2019. “Heavy‐Tailed Prior Distributions for Sequence Count Data: Removing the Noise and Preserving Large Differences.” Bioinformatics 35, no. 12: 2084–2092. 10.1093/bioinformatics/bty895. PMC6581436 30395178

[acel70788-bib-0091] Zurovcova, M. , and W. F. Eanes . 1999. “Lack of Nucleotide Polymorphism in the Y‐Linked Sperm Flagellar Dynein Gene Dhc‐Yh3 of *Drosophila melanogaster* and *D. simulans* .” Genetics 153, no. 4: 1709–1715. 10.1093/genetics/153.4.1709. PMC1460849 10581278

