# Supplementary material for: Exploring the Role of Transposable Elements to Sex Gap in Longevity in Three Drosophila Species
Source: Aging Cell. 2026 Nov 3;25(11):e70788. doi: 10.1111/acel.70788 (PMC13731165; doi:10.1111/acel.70788)
Supplement: Supplementary file 1 [file ACEL-25-e70788-s001.docx]

**SUPPLEMENTARY INFORMATION**

**Figure S1.** Natural variability in SGL in different *Drosophila* species. Histograms displaying the distribution of SGL values calculated as the logarithm of the ratio between male and female median lifespan. **A**) Histogram displaying the distribution of SGL values per population in *D. melanogaster*, *D. simulans*, and *D. suzukii*. UK: United Kingdom, FR: France, FI: Finland, NL: the Netherlands, BR: Brazil, ES: Spain, RS: Serbia. **B**) Histogram displaying the distribution of SGL values per species.

**Figure S2.** PCA analysis plot on normalized counts for genes (left), and TEs (right) in **A)** *D. melanogaster*, **B)** *D. simulans*, and **C)** *D. suzukii*. PCA revealed that PC1 accounts for over 60% of the variance and separates samples by sex across all three Drosophila species. PC2 explained 7-15% of the variance, distinguishing samples by age in *D. melanogaster* and *D. suzukii*, and by strain in *D. simulans*. PCA of TE counts revealed that in *D. melanogaster*, PC1 (43% variance) separates samples by age, while PC2 (21% variance) separates them by sex. In *D. simulans* and *D. suzukii*, PC1 explains 73% and 43% of the variance, respectively, separating samples by strain, while PC2 accounts for 10% and 24% of the variance, respectively, separating samples by age.

**Figure S3. A)** Volcano plots of DEGs between old and young samples in all the species tested. Statistically significant upregulated genes are depicted in red and statistically significant downregulated genes are in blue. The name of the five most upregulated and downregulated genes in each strain are shown. **B)** Barplots showing the number of upregulated (in red) and downregulated (in blue) genes between old and young samples in all the strains and species tested. Results for males are shown in upper panels while results for females are in lower panels.

**Figure S4**. Heatmaps showing the expression of previously-associated ageing genes in all the strains from *D. melanogaster*, *D. simulans*, and *D. suzukii*, averaged across replicates. Colors represent log2 fold changes, with red corresponding to upregulated genes and blue corresponding to downregulated genes. Significant p adjusted values are represented with asterisks.

**Figure S5**. Volcano plots of DE TE families between old and young samples in all the species tested. Statistically significant upregulated TE families are depicted in red and statistically significant downregulated TE families are in blue. The name of the five most upregulated and downregulated TE families in each strain are shown.

**Figure S6**. Upset plots showing the number of shared DE TE families between samples. Highlighted in red are the number of shared DE TE families between all the samples.

**Figure S7**. Volcano plots of DE TE families between old male flies and old female flies in all the species tested. Statistically significant upregulated TE families in old males are depicted in red and statistically significant downregulated TE families in old males are depicted in blue. The name of the five most upregulated and downregulated TE families in each strain are shown.

**Figure S8**. Volcano plots of DETE copies between old and young samples in all the species tested. Statistically significant upregulated TE copies are depicted in red and statistically significant downregulated TE copies are in blue.

**Figure S9**. Upset plots showing the number of shared DE TE copies between males and females from the same strain. Highlighted in red are the number of shared DE TE copies between both samples.

**Figure S10**. Volcano plots of DETE copies between old male flies and old female flies in all the species tested. Statistically significant upregulated TE copies are depicted in red and statistically significant downregulated TE copies are in blue.

**Figure S11**. Circular track plots showing the distribution of TE insertions (in grey) and upregulated TE copies (in red) in the genome of the *D. melanogaster* strains (plots represented in blue), *D. simulans* (orange), and *D. suzukii* (pink). Plots show the biggest scaffolds assembled for simplification. Circular track plots were generated with the *BioCircos* R package using for each strain its genome assembly and a *bed* file containing the coordinates of the annotated TEs and the upregulated TE copies in old males compared to old females.

**Figure S12**. **A**) Crossing scheme for both reciprocal crosses to obtain flies with Y chromosome replacements. “A” represents all autosomes. **B**) SGL values measured as the logarithm of the ratio between male and female median lifespan are shown for A reciprocal crosses. Results for maternal strains are shown in grey (dmgoth101) and black (dmgoth63) while different colours represent generations of introgression from F1 to F5. Dots show mean SGL values and lower and upper whiskers indicate the upper and lower 95% confidence intervals. **C**) Kaplan-Meier survivorship curves for males (left panel, n=150 each generation) and females (right panel, n=150 each generation) in A reciprocal crosses. Colours represent the different generations as in panel B. Dashed lines represent median lifespan values. **D**) SGL values measured as the logarithm of the ratio between male and female median lifespan for B reciprocal crosses. Results for maternal strains are shown in grey (dmgoth101) and black (dmgoth63) while different colours represent generations of introgression from F1 to F5. Dots show mean SGL values and lower and upper whiskers indicate the upper and lower 95% confidence intervals. **E**) Kaplan-Meier survivorship curves for males (left panel, n=150 each generation) and females (right panel, n=150 each generation) in B reciprocal crosses. Colours represent the different generations as in panel D. Dashed lines represent median lifespan values.

**Figure S13**. **A**) Crossing scheme for both reciprocal crosses to obtain flies with Y chromosome replacements. “A” represents all autosomes. **B**) SGL values measured as the logarithm of the ratio between male and female median lifespan are shown for A reciprocal crosses. Results for maternal strains are shown in black (dsgoth31) and grey (dssj27) while different colours represent generations of introgression from F1 to F5. Dots show mean SGL values and lower and upper whiskers indicate the upper and lower 95% confidence intervals. **C**) Kaplan-Meier survivorship curves for males (left panel, n=150 each generation) and females (right panel, n=150 each generation) in A reciprocal crosses. Colours represent the different generations as in panel B. Dashed lines represent median lifespan values. **D**) SGL values measured as the logarithm of the ratio between male and female median lifespan for B reciprocal crosses. Results for maternal strains are shown in black (dsgoth31) and grey (dssj27) while different colours represent generations of introgression from F1 to F5. Dots show mean SGL values and lower and upper whiskers indicate the upper and lower 95% confidence intervals. **E**) Kaplan-Meier survivorship curves for males (left panel, n=150 each generation) and females (right panel, n=150 each generation) in B reciprocal crosses. Colours represent the different generations as in panel D. Dashed lines represent median lifespan values.

**Figure S14**. **A**) On the left, crossing scheme used to obtain male *D. melanogaster* flies for genome size estimation by flow cytometry. The picture illustrates reciprocal crosses between parental strains, indicating the origin of the Y chromosome and autosomal background in the resulting male progeny. On the right, genome size estimates obtained by flow cytometry for males from the parental strains and from the offspring of both reciprocal crosses (n=50). Each point represents an individual measurement, and horizontal lines indicate the median. **B**) The same as in A), but for *D. simulans*. Asterisks indicate level of statistical significance of one-sided Pairwise Wilcoxon tests (* p ≤ 0.05, ** p ≤ 0.01, ***p ≤ 0.001).

**Figure S15**. **A**) Scatter plots of genes comparing F10 and F1 introgression generations of both A and B reciprocal crosses between dmgoth101 and dmsj7 strains. Upregulated genes are shown in red while downregulated genes are shown in blue, under the cutoffs of FDR < 0.05 and log2 fold-change > |1|. **B**) Scatter plots of TE families comparing F10 and F1 introgression generations of both A and B reciprocal crosses between dmgoth101 and dmsj7 strains. Upregulated TE families are shown in red while downregulated TE families are shown in blue, under the cutoffs of FDR < 0.05 and log2 fold-change > |1|.

**Figure S16**. GO analysis comparing F10 and F1 introgression generations of both A and B reciprocal crosses between dmgoth101 and dmsj7 strains.
